# Canadian 24-Hour Movement Guidelines for the Early Years (0–4 years): An Integration of Physical Activity, Sedentary Behaviour, and Sleep

**DOI:** 10.1186/s12889-017-4859-6

**Published:** 2017-11-20

**Authors:** Mark S. Tremblay, Jean-Philippe Chaput, Kristi B. Adamo, Salomé Aubert, Joel D. Barnes, Louise Choquette, Mary Duggan, Guy Faulkner, Gary S. Goldfield, Casey E. Gray, Reut Gruber, Katherine Janson, Ian Janssen, Xanne Janssen, Alejandra Jaramillo Garcia, Nicholas Kuzik, Claire LeBlanc, Joanna MacLean, Anthony D. Okely, Veronica J. Poitras, Mary-Ellen Rayner, John J. Reilly, Margaret Sampson, John C. Spence, Brian W. Timmons, Valerie Carson

**Affiliations:** 1 Healthy Active Living and Obesity Research Group, Children’s Hospital of Eastern Ontario Research Institute, 401 Smyth Road, Ottawa, ON K1H 8L1 Canada; 2 School of Human Kinetics, Faculty of Health Sciences, University of Ottawa, Ottawa, ON K1N 1A2 Canada; 3 Best Start Resource Centre, Health Nexus, Toronto, ON M5G 1Z8 Canada; 4 Canadian Society for Exercise Physiology, Ottawa, ON K1R 6Y6 Canada; 5 School of Kinesiology, University of British Columbia, Vancouver, BC V6T 1Z3 Canada; 6 Department of Psychiatry, Faculty of Medicine, McGill University, Montreal, QC H3A 1A1 Canada; 7 ParticipACTION, Toronto, ON M5S 1M2 Canada; 8 School of Kinesiology and Health Studies, and Department of Community Health and Epidemiology, Queen’s University, Kingston, ON K7L 3N6 Canada; 9 University of Strathclyde, School of Psychological Science and Health, Glasgow, Scotland G1 1QE UK; 10 Public Health Agency of Canada, Ottawa, ON K1A 0K9 Canada; 11 Faculty of Physical Education and Recreation, University of Alberta, Edmonton, AB T6G 2H9 Canada; 12 Montreal Children’s Hospital, Montreal, QC H3H 1P3 Canada; 13 Department of Pediatrics, Faculty of Medicine & Dentistry, University of Alberta, Edmonton, AB T6G 1C9 Canada; 14 Early Start Research Institute, Faculty of Social Sciences, University of Wollongong, Wollongong, NSW 2522 Australia; 15 The Sandbox Project, Toronto, ON M5C 2C5 Canada; 16 Library Services, Children’s Hospital of Eastern Ontario, Ottawa, ON K1H 8L1 Canada; 17 Child Health & Exercise Medicine Program, Department of Pediatrics, McMaster University, Hamilton, ON L8S 4K1 Canada

**Keywords:** Infants, Toddlers, Preschoolers, Adiposity, Motor development, Cognitive development, Public health, Recommendations, Guideline development

## Abstract

**Background:**

The Canadian Society for Exercise Physiology convened representatives of national organizations, research experts, methodologists, stakeholders, and end-users who followed rigorous and transparent guideline development procedures to create the *Canadian 24-Hour Movement Guidelines for the Early Years (0–4 years): An Integration of Physical Activity, Sedentary Behaviour, and Sleep*. These novel guidelines for children of the early years embrace the natural and intuitive integration of movement behaviours across the whole day (24-h period).

**Methods:**

The development process was guided by the Appraisal of Guidelines for Research and Evaluation (AGREE) II instrument. Four systematic reviews (physical activity, sedentary behaviour, sleep, combined behaviours) examining the relationships within and among movement behaviours and several health indicators were completed and interpreted by a Guideline Development Panel. The systematic reviews that were conducted to inform the development of the guidelines, and the framework that was applied to develop the recommendations, followed the Grading of Recommendations Assessment, Development, and Evaluation (GRADE) methodology. Complementary compositional analyses were performed using data from the Canadian Health Measures Survey to examine the relationships between movement behaviours and indicators of adiposity. A review of the evidence on the cost effectiveness and resource use associated with the implementation of the proposed guidelines was also undertaken. A stakeholder survey (*n* = 546), 10 key informant interviews, and 14 focus groups (*n* = 92 participants) were completed to gather feedback on draft guidelines and their dissemination.

**Results:**

The guidelines provide evidence-informed recommendations as to the combinations of light-, moderate- and vigorous-intensity physical activity, sedentary behaviours, and sleep that infants (<1 year), toddlers (1–2 years) and preschoolers (3–4 years) should achieve for a healthy day (24 h). Proactive dissemination, promotion, implementation, and evaluation plans were prepared to optimize uptake and activation of the new guidelines.

**Conclusions:**

These guidelines represent a sensible evolution of public health guidelines whereby optimal health is framed within the balance of movement behaviours across the whole day, while respecting preferences of end-users. Future research should consider the integrated relationships among movement behaviours, and similar integrated guidelines for other age groups should be developed.

**Electronic supplementary material:**

The online version of this article (10.1186/s12889-017-4859-6) contains supplementary material, which is available to authorized users.

## Background

Movement behaviours across the whole day (24-h period) have recently garnered increased interest in public health research and practice. For example, Canada released evidence-informed *Canadian 24-Hour Movement Guidelines for Children and Youth: An Integration of Physical Activity, Sedentary Behaviour, and Sleep* [1] in response to undesirable trends in childhood physical activity [2–4], sedentary behaviour [2–4], and sleep [4–6]. Systematic reviews of studies involving these three topics show desirable movement behaviours (e.g., longer sleep, less sedentary behaviour or screen time, more physical activity) to be beneficially associated with a variety of holistic health indicators in children and youth [7–10]. Subsequent analyses demonstrated consistent evidence of additional health benefits associated with meeting an increased number of movement behaviour guidelines [11–14], supporting their integration in public health messaging. Feedback from stakeholder and end-user groups is supportive of this integrated approach [15, 16].

Recent systematic reviews that focused on children of the early years (0–4 years) and examined the relationships between health indicators and physical activity [17–19], sedentary behaviours [20, 21], sleep duration [22], and movement behaviour combinations [23] all suggest health benefits associated with desirable movement behaviours. Current evidence indicates that 62–84% of Canadian preschoolers (aged 3–4 years) are meeting physical activity guidelines [24–26]; however, only 18–24% meet current screen time recommendations [24–27]. Evidence on toddlers (aged 1–2 years) indicates virtually all meet the physical activity guidelines but only 15% meet screen time guidelines [28]. Concern over the low proportion of children of the early years meeting screen time guidelines [27] is evidenced by recent published statements by the American Academy of Pediatrics [29] and the Canadian Paediatric Society [30]. Several recent reviews have explored potential mechanisms linking excessive screen time with health indicators [20, 31, 32]. There are no previous systematic review-informed Canadian sleep guidelines for the early years [22]. Collectively these findings provide evidence of the importance of all movement behaviours in the early years.

Following the rationale to develop the *Canadian 24-Hour Movement Guidelines for Children and Youth* [1], extending this whole-day approach to movement behaviours to the early years is a natural evolution of this work. Furthermore, evidence from focus group discussions and key informant interviews suggest that stakeholders (e.g., clinicians, practitioners, physical activity knowledge translation groups, researchers) and end-users’ (e.g., pediatricians, parents, early childhood educators) are supportive of a similar approach for children of the early years [33]. Therefore, the purpose of this manuscript is to outline the process and outcomes for the development of the *Canadian 24-Hour Movement Guidelines for the Early Years (0–4 years): An Integration of Physical Activity, Sedentary Behaviour, and Sleep* released by the Canadian Society for Exercise Physiology and partners on November 20, 2017. There are no previously developed Canadian evidence-based guidelines integrating recommendations for all movement behaviours for the early years. These new, integrated recommendations are intended to provide parents, caregivers, health professionals, and policy-makers with guidance on the quality and quantity of physical activity, sedentary behaviour, and sleep in a 24-h period to achieve the greatest health benefits in children 0 – 4 years of age. Throughout this paper the term “movement behaviours” is used to encompass physical activities of all intensities, sedentary behaviours (defined as any waking behaviour characterized by an energy expenditure ≤1.5 metabolic equivalents, while in a sitting, reclining or lying posture [34]), and sleep; thus, conceptualizing movement on a continuum from sleep to high-intensity physical activity [35].

## Methods

### Overall guideline development process

**Fig. 1:**
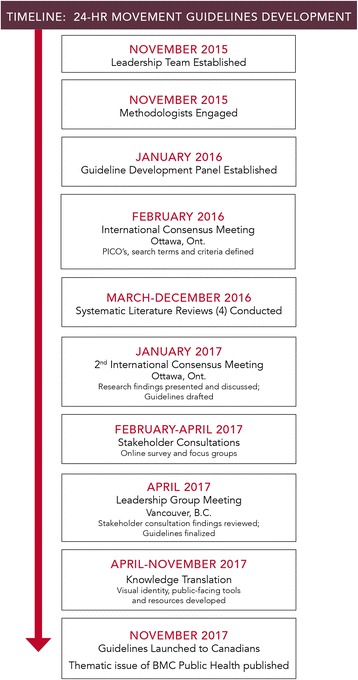
Timelines and sequence of events involved in the development of the *Canadian 24-Hour Movement Guidelines for the Early Years (0–4 years): An Integration of Physical Activity, Sedentary Behaviour, and Sleep*

The process used to develop the *Canadian 24-Hour Movement Guidelines for the Early Years (0–4 years)* followed the 15-stage framework described in detail by Tremblay and Haskell [36]. The process included the application of the Appraisal of Guidelines for Research and Evaluation (AGREE) II instrument [37–40] from the outset as well as the early engagement of guideline development methodologists, and benefitted from the significant experience and learning from earlier guideline development, dissemination, and implementation efforts. Fig. 1 provides a summary of the timelines and sequence of events involved in the development of the guidelines.

In November 2015, a Leadership Committee was formed that included the project principal investigators, representatives from each of the funding partners (Canadian Society for Exercise Physiology [CSEP]; Healthy Active Living and Obesity Research Group [HALO] at the Children’s Hospital of Eastern Ontario Research Institute; University of Alberta; and ParticipACTION), methodologists, and support staff. In August 2016, the Public Health Agency of Canada (PHAC) provided support for the guideline development and also joined the Leadership Committee. The Leadership Committee met monthly to provide oversight, strategic direction, fiscal accountability, and attentiveness to AGREE II criteria. Subsets of the Leadership Committee met as required to ensure the project advanced efficiently. In January 2016, a Guideline Development Panel (GDP) was formed with members including research experts, stakeholder groups, knowledge users, international collaborators, methodology consultants, parents, and project managers (Table 1).

**Table 1:** Guideline Development Panel

Panel member	Affiliation	Role	Conflict of interest declaration
Research experts and credentials			
Kristi Adamo, PhD	Associate Professor, University of Ottawa (Canada)	PA and SB content expert, systematic review author	none
Salome Aubert	doctoral student, University of Ottawa (Canada)	PA and SB content expert, systematic review author	none
Valerie Carson, PhD	Associate Professor, University of Alberta (Canada)	compositional analyses leader, PA and SB content expert, Leadership Committee, Steering Committee, Surveillance Sub-Committee, systematic review author	none
Jean-Philippe Chaput, PhD	Research Scientist, HALO, CHEO RI (Canada)	sleep, PA, and SB content expert, Leadership Committee, Steering Committee, Surveillance Sub-committee, systematic review author	none
Guy Faulkner, PhD	Professor and CIHR-PHAC Chair in Applied Public Health, University of British Columbia (Canada)	PA and SB content expert, stakeholder consultation (focus groups author)	none
Gary Goldfield, PhD	Senior Scientist, HALO, CHEO RI (Canada)	PA and SB content expert, systematic review author	none
Reut Gruber, PhD	Professor, McGill University; Director, Attention Behaviour and Sleep Lab, Douglas Mental Health University Institute (Canada)	sleep content expert, systematic review author	Husband on ACSM Board of Directors 2010–2016 (ACSM produced clinical guidelines and position stands for sleep medicine field); received several grants as a Principal Investigator to investigate the interplay between sleep, nutrition and PA in children and developed an intervention program to target this interplay, expects to publish.
Ian Janssen, PhD	Professor and Canada Research Chair in Physical Activity and Obesity, Queen’s University (Canada)	PA and SB content expert, Surveillance Sub-Committee, systematic review author	none
Nicholas Kuzik	doctoral student, University of Alberta (Canada)	combined movement behaviour content expert, systematic review author, Leadership Committee	none
Joanna MacLean, PhD, MD, FRCPC	paediatric respirologist and sleep medicine specialist; Associate Professor, University of Alberta (Canada)	sleep content expert, systematic review author	none
John Spence, PhD	Professor and Vice-Dean of Physical Education and Recreation, University of Alberta (Canada)	PA and SB content expert, systematic review author	none
Brian Timmons, PhD	Associate Professor and Canada Research Chair in Child Health and Exercise Medicine, McMaster University (Canada)	PA and SB content expert, systematic review author	none
Mark Tremblay, PhD	Director, HALO, and Senior Scientist CHEO RI (Canada)	Chair, PA and SB content expert, Leadership Committee, Surveillance Sub-Committee, Steering Committee, systematic review author, dissemination and implementation, evaluation	none
Stakeholder groups and knowledge users			
Louise Choquette	bilingual health promotion consultant, Health Nexus (Canada)	invited representative (Health Nexus), early years expert	none
Mary Duggan, CAE	Manager, CSEP (Canada)	CSEP representative, Leadership Committee, Steering Committee, dissemination and implementation, evaluation	none
Katherine Janson	Director of Communications and Public Affairs, ParticipACTION (Canada)	invited representative (ParticipACTION), creative development and marketing, Leadership Committee	none
Claire LeBlanc, MD, FRCPC	paediatric rheumatologist and sport medicine physician, Montreal Children’s Hospital (Canada)	invited representative (Canadian Pediatric Society,) early years, PA, SB, and sleep content expert	none
Mary-Ellen Rayner	Chief Partnerships and Communications Officer, The Sandbox Project	invited representative (The Sandbox Project), early years, PA, and SB content expert	none
International collaborators			
Xanne Janssen, PhD	Postdoctoral Fellow, University of Strathclyde (Scotland)	PA and SB content expert, international representative, systematic review author	none
Anthony Okely, PhD	Professorial Fellow and Director, Early Start Institute, University of Wollongong (Australia)	early years, SB, and PA content expert, international representative, systematic review author	Received funding as a consultant from Foxtel to advise on PA interstitial as part of their preschool television programs
John Reilly, PhD	Professor, University of Strathclyde (Scotland)	early years, PA and SB content expert, international representative, systematic review author	none
Methodology consultants and project management			
Casey Gray, PhD	Project Manager, HALO, CHEO RI (Canada)	PA and SB content expert, Leadership Committee, Steering Committee, systematic review author, evaluation	none
Alejandra Jaramillo Garcia	Global Health and Guidelines Division, PHAC (Canada)	AGREE II and GRADE methodological consultant, Steering Committee, systematic review author	none
Veronica Poitras, PhD	Clinical Research Officer, Canadian Agency for Drugs and Technologies in Health (Canada)^a^	PA and SB content expert, Leadership Committee, Steering Committee, Surveillance Sub-Committee, systematic review author	none
Margaret Sampson, PhD	Manager, Library Services, Children’s Hospital of Eastern Ontario (Canada)	methodology expert, research librarian, systematic review author	none

*ACSM* American College of Sports Medicine, *AGREE* Appraisal of Guidelines for Research and Evaluation; *CAE* Certified Association Executive, *CHEO RI* Children’s Hospital of Eastern Ontario Research Institute; *CIHR* Canadian Institutes of Health Research, *CSEP* Canadian Society for Exercise Physiology, *FRCPC* Fellow of the Royal College of Physicians of Canada, *GRADE* Grading of Recommendations Assessment, Development, and Evaluation, *HALO* Healthy Active Living and Obesity Research Group, *PA* physical activity, *PHAC* Public Health Agency of Canada, *SB*, sedentary behaviour

^a^Veronica Poitras was a Research Manager (HALO, CHEO RI) during the conduct of the systematic reviews and preparation of the initial draft of the guidelines

The GDP met in February 2016, for a two-day meeting. The objectives of this initial meeting were to provide an overview of the guideline development process, responsibilities, and timelines; introduce the methodology consultants and explain their responsibilities; hear from international delegates about other countries’ guideline processes and the potential for harmonization and avoiding duplication of efforts; finalize the systematic review parameters; finalize the search strategies for the systematic reviews; discuss and set timelines for the systematic reviews; and initiate discussions regarding knowledge translation, dissemination, and evaluation. In accordance with the GRADE handbook [41], the group also identified and prioritized the health outcomes/indicators for each of the systematic reviews, with a focus on health outcomes/indicators valued by the individuals who will be applying these guidelines (e.g., parents, early childhood educators, health professionals). In the context of paediatrics, health outcomes (e.g., disease manifestations, mortality) are uncommon, so for the purposes of this manuscript the term “health indicator” will be used.

### Systematic reviews

Though the initial GDP meeting was funded by a Canadian Institutes of Health Research (CIHR) grant to update the previous early years sedentary behaviour guidelines [27], the Leadership Committee agreed that the effort must adhere to the whole-day approach taken with the new *Canadian 24-Hour Movement Guidelines for Children and Youth* [1], and additional funding sources were discussed to support this approach. Consequently, four systematic reviews were required. A brief overview of each systematic review is provided below, with full details available elsewhere in this special supplement [18, 21–23]. Reviewers systematically searched online databases for articles on apparently healthy children, including those with obesity, but excluding papers specifically targeting children with known disease, disability or impairments. The early years were defined as ages 0–4 years and further subdivided into infants (<1 year), toddlers (1–2 years), and preschoolers (3–4 years). The quality of evidence in each systematic review was assessed by indicator, study design, and age group (where possible), using the Grading of Recommendations Assessment, Development, and Evaluation (GRADE) framework [42, 43].

The first systematic review examined the relationships between objectively and subjectively measured physical activity and health indicators in the early years [18], updating and building on an earlier systematic review used to inform *Canadian Physical Activity Guidelines for the Early Years* [26, 44]. As detailed in the international Prospective Register of Systematic Reviews (PROSPERO; Registration no. CRD42016035937), the Population, Intervention, Comparator, and Outcome (PICO) parameters [45] included apparently healthy children aged 1 to <60 months; objectively and subjectively measured physical activity; various volumes, durations, frequencies, patterns, types, and intensities of physical activity; and both critical (adiposity, motor development, psychosocial health, cognitive development, fitness) and important (bone and skeletal health, cardiometabolic health, and risks/harm) health indicators [18]. Note that according to GRADE, outcomes rated as “critical” are those that are considered essential for decision-making; these are weighted most heavily in the process of moving from the evidence to the guideline recommendations (see *GRADE evidence to decision framework: summary* below). “Important” health indicators were also identified by the GDP but given lower weighting through the evidence to decision process.

Updating and extending the earlier systematic review that LeBlanc et al. [46] conducted for the *Canadian Sedentary Behaviour Guidelines for the Early Years* [27], the second systematic review examined relationships between sedentary behaviour and health indicators in the early years (PROSPERO Registration no. CRD42016035270) [21]. PICO parameters included the population of apparently healthy children aged 1 to <60 months; interventions and comparators were durations, patterns, and types of sedentary behaviours (e.g., seated watching television, playing on the computer, reading, eating, travelling in a car); and both critical (adiposity, motor development, psychosocial health, cognitive development) and important (bone and skeletal health, cardiometabolic health, fitness, and risks/harms) health indicators [21].

The objective of the third systematic review was to examine the associations between sleep duration and health indicators in children of the early years (PROSPERO Registration no. CRD42016040096) [22]. The review included apparently healthy children aged 1 to <60 months; interventions and comparators of various sleep durations; and both critical (adiposity, emotional regulation, cognitive development, motor development, growth) and important (cardiometabolic health, sedentary behaviour, physical activity, quality of life/well-being, risks/injuries) health indicators [22].

The fourth systematic review examined combinations of two or more movement behaviours and the associations with health indicators in children of the early years (PROSPERO Registration no. CRD42015015493) [23]. The PICO parameters included the population of apparently healthy children aged 1 to <60 months; intervention/exposure (combination of ≥2 movement behaviours [i.e., sleep and sedentary behaviour; sleep and physical activity; sedentary behaviour and physical activity; and sleep, sedentary behaviour, and physical activity]); comparator (various levels and combinations of movement behaviours); and indicators (critical: adiposity, motor development, psychosocial health/emotional regulation, cognitive development, fitness, and growth; important: bone and skeletal health, cardiometabolic health, and risks) [23].

### Compositional analyses

Research on movement-related behaviours and the resultant public health guidelines have typically taken a segregated rather than integrated approach. Not surprisingly, the evidence base is similarly constructed. While three of the systematic reviews outlined above provide a comprehensive assessment of the relationships between individual movement behaviours (i.e., sleep, sedentary behaviour, physical activity) and indicators of health, only the review by Kuzik et al. [23] examined evidence in relation to combinations of two or more movement behaviours. Examining the combinations of movement behaviours that constitute the complete 24-h period is not common and presents inherent analytical challenges [47, 48]. Because the constituent parts (sleep, sedentary behaviour, physical activity) saturate the entire 24-h period, a change in any behaviour must be done at the expense of one of the other behaviours, making the variables time-dependent and constitutionally collinear. To address this reality, assess the legitimacy of the whole-day approach to health promotion in the early years, and help inform the new integrated guidelines, complementary compositional data analyses [47, 49] were conducted using data from the Canadian Health Measures Survey (CHMS) [50].

The specific objectives of the compositional analyses were twofold: (1) to explore the combined associations of the composition of sleep duration, sedentary time, light-intensity physical activity (LPA), and moderate- to vigorous-intensity physical activity (MVPA) with adiposity indicators, and (2) to explore the association of the time spent in sleep, sedentary behaviour, LPA, and MVPA with adiposity indicators relative to the time spent in the other behaviours in a representative sample (*n* = 552) of Canadian preschool-aged children from the CHMS [51]. Sedentary time, LPA, and MVPA were measured with Actical accelerometers (Philips Respironics, Bend, Oregon, USA); sleep duration was measured by parental report. Height and body mass (to determine body mass index [BMI] z-scores based on World Health Organization growth standards) and waist circumferences (WC) were directly measured following standard procedures [52–54]. Compositional data analyses were used to examine the cross-sectional associations. For complete details on the sample, measurement procedures and compositional analyses see Carson et al. [51]. These compositional analytic procedures can be used as a blueprint for future research to examine the associations of multiple movement behaviours and other health indicators beyond adiposity.

### Additional considerations from GRADE

The GRADE process uses several sources of information in a systematic and transparent fashion to inform guideline recommendations. These factors include quality of the evidence (i.e., risk of bias, inconsistency, indirectness, imprecision, publication bias), balance of benefits and harms, end-user preferences and values, resource implications, feasibility, acceptability, and equity issues. This collection of information is used to inform the direction (i.e., for or against) and the strength (i.e., strong or conditional/weak) of the recommendation.

The quality of the evidence was assessed and reported in the systematic reviews [18, 21–23]. The balance of benefits and harms was also informed by the systematic reviews as well as by detailed discussions and eventual consensus by the GDP. End-user preferences and values, resource implications, feasibility, acceptability, and equity issues were assessed through the stakeholder survey, key informant interviews, and focus groups (described in *Guidelines recommendations and stakeholder consultations* section). To further explore resource requirements (costs), a review of the evidence on cost and resource use related to 24-h movement behaviours was conducted. However, no evidence was found that met the inclusion criteria.

### Guidelines recommendations and stakeholder consultations

The second meeting of the GDP was held in January 2017. The objectives of this three-day meeting were to review, discuss, debate, and interpret findings from systematic reviews and compositional analyses; review results of cost-effectiveness/resource use analysis; craft individual components of the movement behaviour guidelines; create 24-h integrated movement behaviour guidelines; identify research gaps; and plan the launch, dissemination, promotion, and evaluation activities. Draft guideline recommendations were created by the GDP based on the overall balance between the possible benefits and harms of various levels of physical activity, sedentary behavior, and sleep; stakeholder and end-user preferences and values related to these movement behaviours; and considerations related to feasibility, accessibility, resource use, and equity. The draft guidelines were translated into French and back-translated for verification. All GDP members approved the draft guidelines for the stakeholder consultations.

A cross-sectional survey (see Additional file 1 for complete survey in English and French) was developed to gather stakeholder and end-user feedback on (1) the content and format of the draft guidelines, (2) elements of importance to the GRADE Evidence to Decision Framework (i.e., how much end-users value the outcomes, the magnitude of the resource use requirements/perceived incremental costs associated with implementing the guidelines, equity, acceptability, and feasibility of implementing the guidelines) [55], and (3) suggestions regarding key intermediaries to implement and activate the guidelines. Following approval from the Children’s Hospital of Eastern Ontario Research Institute’s Research Ethics Board, the survey was created online using REDCap (Research Electronic Data Capture) [56] software and was open from March 24 to April 18, 2017. Participants were recruited via a snowball sampling procedure, initiated through GDP distribution networks. Data were imported into Excel (Microsoft Corporation, Seattle, Washington, USA) for analysis of closed- and open-ended responses. Descriptive statistics were calculated to summarize participant characteristics and closed-ended feedback. Open-ended feedback was synthesized qualitatively, using thematic analyses whereby research staff read through the full transcripts of participant responses and independently identified common themes that emerged from the data. Independent assessments were discussed among the assessors until agreement on a final set of themes was achieved.

In addition to the online stakeholder survey, a series of focus groups and key informant interviews were completed to examine stakeholders’ (experts in pediatric and family medicine, physical activity knowledge translation, and research) and end-users’ (parents and early childhood educators) perceptions of the draft guidelines [33]. Ethics approval for these consultations was obtained from the Research Ethics Boards of the University of British Columbia and the Children’s Hospital of Eastern Ontario Research Institute. Stakeholders (*n* = 10) engaged in telephone interviews and end-users (*n* = 92) participated in focus groups (*n* = 14) to discuss perceived clarity and need for the guidelines, potential barriers to implementation, identification of credible messengers, and methods for dissemination of the guidelines. Audio-recordings from the focus groups and interviews were transcribed verbatim and thematic analysis was conducted consistent with that reported by Faulkner et al. [16]. Full details on the methodology are available elsewhere [33].

A sub-committee of the GDP reviewed summaries of the stakeholder survey, focus group and interview results, and revised the guidelines based on the feedback, ensuring changes remained true to the available evidence base. The revised guidelines were circulated to the entire GDP for comment and final revisions. Consensus was achieved on the final guidelines. Revisions were translated to finalize the French version.

### Dissemination, implementation and evaluation plans

The release of the *Canadian 24-Hour Movement Guidelines for Children and Youth* [1] marked the initiation of a paradigm shift away from consideration of isolated behaviours towards a “whole-day matters” approach. This shift created opportunity for the redevelopment of guideline dissemination and implementation practices [57]. In addition to traditional passive dissemination strategies (e.g., website posts, distribution of print resources), additional efforts were made to implement and activate the child and youth guidelines. An iconic visual identity was created, a pseudo-set (sweat, step, sleep, sit) was established and an interactive web experience was created called “build your best day” (www.buildyourbestday.com). Members of the GDP, with additional members from CSEP and ParticipACTION (Canadian not-for-profit organization promoting physical activity in Canada), formed a sub-committee (Guideline Implementation and Activation Committee) to facilitate strategic and proactive dissemination, promotion, and implementation of the new early years guidelines, building off the work completed for the children and youth guidelines, and maintaining the same “look and feel”. The harmonized visual identity was used in the preparation of materials, tools and resources, both digital and print, for the release of the *Canadian 24-Hour Movement Guidelines for the Early Years*. The Guideline Implementation and Activation Committee also developed an integrated marketing and communications plan for sustained dissemination and implementation following the guideline launch. Finally, the work of the sub-committee responsible for evaluation of the child and youth guidelines was expanded to include assessment of the impact of the dissemination and implementation efforts of the guidelines for the early years.

The guideline development process in Canada instigated a similar process in Australia. Leveraging the background work done in Canada, and guided by the GRADE “adolopment” procedures [58], the *Australian 24-Hour Movement Guidelines for the Early Years* were prepared [59] and concurrent launch plans were coordinated.

### Research gaps and surveillance recommendations

Research gaps were identified and recorded throughout the guideline development process (e.g., systematic reviews, guideline meetings, sub-group discussions). The new paradigm of the 24-h movement guidelines requires earlier surveillance measures to be reconsidered, with a shift from an individual behaviour focus to the combination or composition of the behaviours.

To make recommendations in this regard a Surveillance Sub-committee of the GDP, with additional members with extensive movement behaviour surveillance experience, convened via teleconferences to discuss and develop preliminary recommendations for the monitoring and surveillance of the new 24-h guidelines, following an approach similar to what was done for the child and youth guidelines [1].

Four independent reviewers were contracted to conduct an AGREE II assessment on the entire guideline development process using the guideline materials and systematic reviews [37–40]. All of the materials presented in this special issue of *BMC Public Health* were provided to the independent assessors.

## Results

### Overall guideline development process

The guideline development process successfully adhered to the framework outlined by Tremblay and Haskell [36]. Throughout the process, methodologists on the GDP familiar with AGREE II [37–40] and GRADE [42, 43, 55] provided advice and kept detailed records of discussions and decisions to help inform the guideline recommendations and the Evidence to Decision Framework [55]. The Leadership Committee and its various sub-committees met in person or by teleconference more than 50 times in the course of the guideline development process. Full GDP meetings were held in Ottawa, Canada in February 2016 and January 2017, with additional correspondence done through email.

### Systematic reviews

A brief summary of the findings of each systematic review is provided below, with detailed results available elsewhere in this special issue of *BMC Public Health* [18, 21–23]. Because of significant heterogeneity in a variety of variables, meta-analyses could not be performed for most indicators in the systematic reviews, so narrative syntheses were predominantly employed. Collectively, 34,566 titles and abstracts were screened and 271 papers were included in the systematic reviews.

#### Physical activity and health indicators

Ninety-six studies (71,291 unique participants from 36 countries) were included in the review on physical activity and health indicators in the early years [18]. Study designs included randomized controlled trials (*n* = 8), clustered randomized controlled trials (*n* = 4), non-randomized interventions (*n* = 9), cross-over trials (*n* = 3), longitudinal (*n* = 7), longitudinal with additional cross-sectional analyses (*n* = 5), case-control (*n* = 4), case cross-over (*n* = 1), and cross-sectional (*n* = 55). One small meta-analysis (four studies, 1100 participants) was conducted examining adiposity as a health indicator; otherwise narrative syntheses were employed. Physical activity was consistently associated with improved motor and cognitive development as well as psychosocial and cardiometabolic health in randomized and non-randomized intervention studies, and with favourable motor development, fitness, and bone and skeletal health in observational studies. Light- and moderate-intensity physical activity were not consistently associated with any health indicators, whereas moderate- to vigorous-intensity, vigorous-intensity, and total physical activity were consistently favourably associated with multiple health indicators. Across study designs, consistent favourable associations with health indicators were observed for different types of physical activity, including active play, aerobic activity, dance, prone position (infants; <1 year), and structured/organized activities. For toddlers and preschoolers, the most favourable frequency and duration of physical activity were unclear, however, more physical activity appeared better for health. For infants, ≥30 min/day of the prone position, or “tummy time”, was most favourably associated with health indicators. The quality of the evidence ranged from “very low” to “high” and the majority of evidence was in preschool-aged children (3–4 years).

#### Sedentary behaviour and health indicators

A total of 96 studies (195,430 participants from 33 countries) were included in the sedentary behaviour systematic review [21]. Study designs included randomized controlled trials (*n* = 1), case-control (*n* = 3), longitudinal (*n* = 25), longitudinal with additional cross-sectional analyses (*n* = 5), and cross-sectional (*n* = 62). Associations between objectively measured total sedentary time and indicators of adiposity and motor development were predominantly null; associations between screen time and indicators of adiposity, motor or cognitive development, and psychosocial health were primarily unfavourable or null. Associations between reading/storytelling and indicators of cognitive development were favourable or null. Associations between time spent seated (e.g., in car seats or strollers) or in the supine position, and indicators of adiposity and motor development, were primarily unfavourable or null. The quality of evidence ranged from “very low” to “moderate” across study designs and health indicators.

#### Sleep and health indicators

The systematic review on sleep duration and health indicators in the early years [22] included 69 studies (62 unique samples; 148,524 unique participants from 23 countries). The study designs included randomized trials (*n* = 3), non-randomized interventions (*n* = 1), longitudinal studies (*n* = 16), cross-sectional studies (*n* = 42), and longitudinal studies that also reported cross-sectional analyses (*n* = 7). Sleep duration was assessed by parental report in 70% of studies (*n* = 48) and was measured objectively (or both objectively and subjectively) in 30% of studies (*n* = 21). In general, shorter sleep duration was associated with higher adiposity, poorer emotional regulation, impaired growth, more screen time, and higher risk of injuries. The evidence related to indicators of cognitive development, motor development, physical activity, and quality of life/well-being was less clear, with no consistent associations. The quality of evidence ranged from “very low” to “high” across study designs and health indicators.

#### Combined movement behaviours and health indicators

The systematic review that examined associations among combinations of movement behaviours and health indicators in children of the early years [23] included 10 studies (7549 participants from 5 countries). Study designs included cluster randomized controlled trials (*n* = 3), non-randomized intervention (*n* = 1), cross-sectional (*n* = 4), and longitudinal (*n* = 2). Across study designs the most ideal combinations of sedentary behaviour and physical activity (i.e., combinations of movement behaviours hypothesized to be beneficial for health, based on research conducted in populations aged ≥5 years; e.g., decreased sedentary behaviour, high physical activity) were favourably associated with motor development and fitness among preschool-aged children; both favourably and not associated with adiposity among toddlers and preschool-aged children; and not associated with growth among toddlers and preschool-aged children. The most ideal combinations of sleep and sedentary behaviour were favourably associated with adiposity among infants and toddlers. The quality of evidence ranged from “very low” to “moderate”. These data indicate that ideal combinations of physical activity, sedentary behaviour and sleep may be important for health in the early years.

Overall, the reviews showed that a need exists for better quality studies with stronger research designs, especially those that can provide information on dose-response relationships, if they exist. Furthermore, they identified a need for future research to determine the ideal distribution and pattern of daily movement behaviours (physical activity, sedentary behaviour, sleep) for optimal health throughout the early years.

### Compositional analyses

Complete data for cross-sectional compositional analyses were available on 552 participants aged 3–4 years from cycles 2 and 3 (2009–2013) of the CHMS [51]. The average age of the sample was 3.5 years and was balanced between males and females (49.2% female). On average, participants spent 30.9% of the 24-h period sedentary, 15.9% in LPA, 4.5% in MVPA, and 48.7% in sleep. The two variables with the highest co-dependence were sedentary time and sleep duration and the two variables with the lowest co-dependence were sedentary time and MVPA. The composition of movement behaviours was significantly associated with BMI z-scores but not with WC. The time spent in sleep, sedentary behaviour, LPA, or MVPA relative to the other behaviours was not significantly associated with the adiposity indicators. This study was the first to use compositional analyses to examine associations of all movement behaviours with adiposity indicators in preschool-aged children. The overall composition of movement behaviours, rather than any single movement behaviour in isolation, appears important for healthy BMI z-scores in preschool-aged children. However, future research, especially experimental research, is needed to determine the optimal movement behaviour composition that should be promoted in this age group and for younger children aged 0–2 years.

### Guideline recommendations and stakeholder consultations

The draft guidelines are available in Additional file 1 as part of the stakeholder survey.

#### Stakeholder survey

*Demographics*: Data from 695 stakeholders and end-users were collected by the online survey; missing data ranged from 130 to 287 per closed-ended item. There were participants from all provinces and territories except the Northwest Territories and Nunavut; the greatest proportion were employed in Ontario (51.7%), followed by Western Canada (36.7%), the Maritimes (3.0%), Quebec (2.7%), and Yukon Territory (0.2%). In addition, 1.2% indicated their work was national in scope and 4.5% worked outside of Canada. By sector, participants were primarily associated with physical activity/fitness (22.9%), public health (16.2%), healthcare (14.2%), education (12.4%), and research (10.2%).

*Content and format of the Guidelines*: A complete summary of the stakeholder survey results is provided in Table 2. Participants agreed with the content and format of the draft title of the guidelines, the preamble, and the guidelines (combined “Strongly Agree” and “Somewhat Agree”: 89.1, 96.2 and 96.1%, respectively), and agreed that the title, preamble, and guidelines were clearly stated (combined “Strongly Agree” and “Somewhat Agree”: 94.2, 96.5, and 98.9%, respectively). A minority of participants suggested additions or changes. Among the feedback received on the title, the most frequent comments suggested the title was too long, the target audience was unclear (i.e., practitioners versus general population), and the early years age range needed to be made explicit. Feedback regarding the preamble suggested it was too long, the target audience was unclear, and the language level was too high if intended for general populations. There was limited feedback provided on the guidelines themselves, and there was no consistent pattern among comments received.

**Table 2:** Summary results of stakeholder survey

Question	Strongly Agree n (%)	Somewhat Agree n (%)	Neither Agree Nor Disagree n (%)	Somewhat Disagree n (%)	Strongly Disagree n (%)	Total Responses n
The Title is clearly stated.	339 (60.0%)	193 (34.2%)	19 (3.4%)	13 (2.3%)	1 (0.2%)	565
Do you agree with the Title?	303 (54.1%)	196 (35.0%)	36 (6.4%)	22 (3.9%)	3 (0.5%)	560
The Preamble is clearly stated.	322 (71.4%)	113 (25.1%)	9 (2.0%)	7 (1.6%)	0 (0.0%)	451
Do you agree with the Preamble?	339 (75.3%)	94 (20.9%)	10 (2.2%)	7 (1.6%)	0 (0.0%)	450
The 24-Hour Guidelines are clearly stated.	341 (78.0%)	87 (20.0%)	5 (1.1%)	4 (1.0%)	0 (0.0%)	437
Do you agree with the 24-Hour Guidelines?	327 (74.8%)	93 (21.3%)	12 (2.7%)	5 (1.1%)	0 (0.0%)	437
Evidence to Decision Framework						
	Yes			No		
Are the 24-Hour Guidelines important to you? (priority)	409 (95.8%)			18 (4.2%)		
	Always	Frequently	Occasionally	Seldom	Never	
Would you use the Preamble? (acceptability)	98 (21.4%)	178 (38.8%)	142 (30.9%)	32 (7.0%)	9 (2.0%)	
Would you use the 24-Hour Guidelines? (acceptability)	141 (32.9%)	198 (46.2%)	73 (17.0%)	11 (2.6%)	6 (1.4%)	
	Much More Useful	More Useful	Neutral	Less Useful	Much Less Useful	
In comparison to separate physical activity, sedentary behaviour and sleep guidelines, do you find these 24-Hour Guidelines... (acceptability)	119 (27.8%)	216 (50.5%)	87 (20.3%)	4 (0.9%)	2 (0.5%)	
	Very Easy	Somewhat Easy	Neither Easy Nor Difficult	Somewhat Difficult	Very Difficult	
How easy or difficult would you find using the 24-Hour Guidelines? (feasibility)	175 (41.0%)	188 (44.0%)	41 (9.6%)	22 (5.2%)	1 (0.2%)	
	Strongly Agree	Somewhat Agree	Neither Agree Nor Disagree	Somewhat Disagree	Strongly Disagree	I Don’t Know
The costs for you to use, or your organization to implement, the 24-Hour Guidelines are likely to be small or negligible compared to not using the Guidelines. (resource use)	143 (35.0%)	122 (29.8%)	55 (13.4%)	12 (2.9%)	5 (1.2%)	27 (6.6%)
The benefits of using the 24-Hour Guidelines are likely to outweigh the costs. (perceived incremental cost-benefit ratio)	211 (51.7%)	120 (29.4%)	47 (11.5%)	3 (0.7%)	1 (0.2%)	26 (6.4%)
Following the 24-Hour Guidelines is likely to benefit all population groups equally, irrespective of gender, race, ethnicity, or the socioeconomic status of the family. (equity)	233 (57.1%)	117 (28.7%)	20 (4.9%)	22 (5.4%)	5 (1.2%)	11 (2.7%)

*GRADE Evidence to Decision Framework*: There was high agreement (>60% was considered high agreement) among participants that the 24-h guidelines are a priority for them (95.8%). There was also high agreement that implementing the guidelines would be feasible (85.0%), acceptable (79.1%), useful (78.3%), cost-effective (64.8%), and equitable across population groups (85.8%). An additional item prompted participants to judge the incremental cost relative to the net benefit of implementing the guidelines; most (81.1%) indicated that the benefits of using the guidelines would likely outweigh the costs.

Open-ended response options were available for participants who wished to explain or elaborate on their responses to closed-ended items for items assessing acceptability, resource use, perceived incremental cost-benefit ratio, and equity. Fifty-seven of the 112 participants who provided feedback to the acceptability item, “Would you use the 24-Hour Guidelines?” responded favourably. Thirty participants provided favourable or neutral feedback and also suggested an addition or change to some element of the guidelines, most commonly requesting examples or added descriptions. Fourteen respondents provided negative feedback and 11 indicated the guidelines were not relevant in their work or their personal life. Fifty-five participants elaborated on their responses to the resource use item. Of those, 10 indicated the cost of using the guidelines would be small to negligible, 4 indicated the cost would be difficult to manage, and 13 described the costs but did not indicate if it would be easy or difficult to absorb into their operating budget. Other responses suggested respondents did not understand the question. Forty participants provided written feedback on the perceived incremental cost-benefit ratio item. Among them, 19 provided favourable responses, one responded that there would be no benefit and only cost, and 19 participants did not provide clear feedback. Fifty-seven participants expanded on their responses to the equity item. Eleven indicated that following the guidelines would benefit all groups equally while two disagreed, expressing a belief that socioeconomic status would moderate the relationship between guideline adherence and any expected health indicators. Thirty-three participants described barriers or facilitators to implementation and 11 did not answer the question.

*Implementation and activation of the guidelines*: Stakeholders (227 total responses; some named multiple intermediaries) indicated childcare providers (176), parents (173), and health care providers (107) were the most important intermediaries to target for implementation and activation of the guidelines. The most endorsed modalities for supporting intermediaries to implement and activate the guidelines (125 total responses) included training opportunities such as prenatal workshops as well as pre-service and in-service training (*n* = 55), and products such as “toolboxes” or posters, with explanations and examples of how to adopt ad activate the guidelines (*n* = 43).

#### Focus groups and key informant interviews

The focus groups and key informant interviews reinforced the findings from the stakeholder survey, that the proposed guidelines were very well received by both stakeholders and end-users [33]. A clear need for such integrated guidelines was identified and most believed the guidelines were achievable. Several potential barriers to uptake were identified including low awareness of current guidelines and ‘daily challenges’ (such as the allure of screen time, lack of time, competing priorities, and challenges in the context of shifting social norms). A range of methods and messengers of dissemination were recommended by focus group participants and key informants. Health care and child care settings were the most frequently cited locations for dissemination and physicians and early childhood educators were the most common suggestions for messengers. Results suggest that going forward it will be important to dedicate appropriate support and funding toward dissemination efforts in order to reach end-users, particularly parents, health care providers, and early childhood educators.

#### Revisions to draft guidelines

Following the Guideline Revision Meeting, three changes were made that altered the content of the preamble: 1) in the description of the groups for whom the guidelines are relevant, the GDP decided that replacing “race and ethnicity” with “cultural background” (adopted in the Australian guidelines) would better indicate the diversity of populations to whom the guidelines apply; 2) in the description of the context of children’s activities it was decided that all adults have a role in helping children of the early years to meet the guidelines and therefore, “parents and caregivers” was replaced with “adults”; and 3) in describing the expected balance of benefits and unfavourable outcomes associated with following the guidelines, the term “risk” was replaced with “harm” for accuracy.

**Fig. 2:**
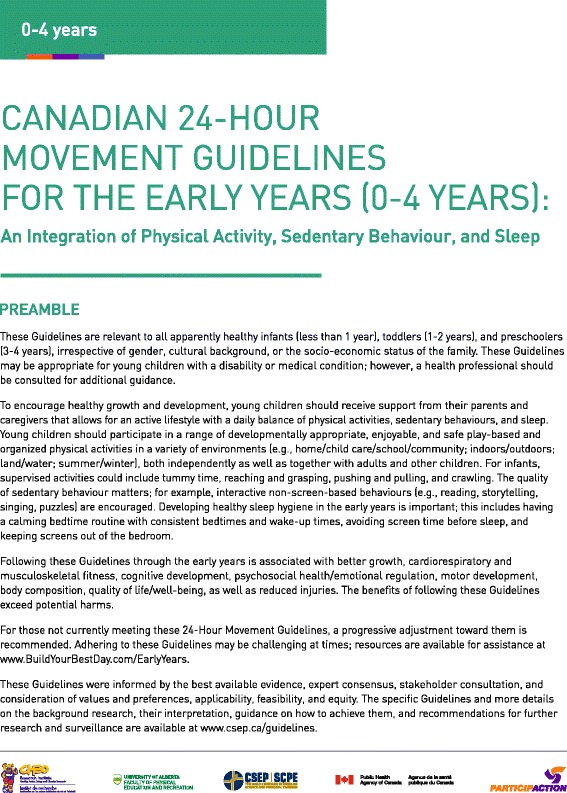
Canadian 24-Hour Movement Guidelines for the Early Years (0–4 years): *An Integration of Physical Activity, Sedentary Behaviour, and Sleep* – English Preamble. © Canadian Society for Exercise Physiology, 2017. All rights reserved

**Fig. 3:**
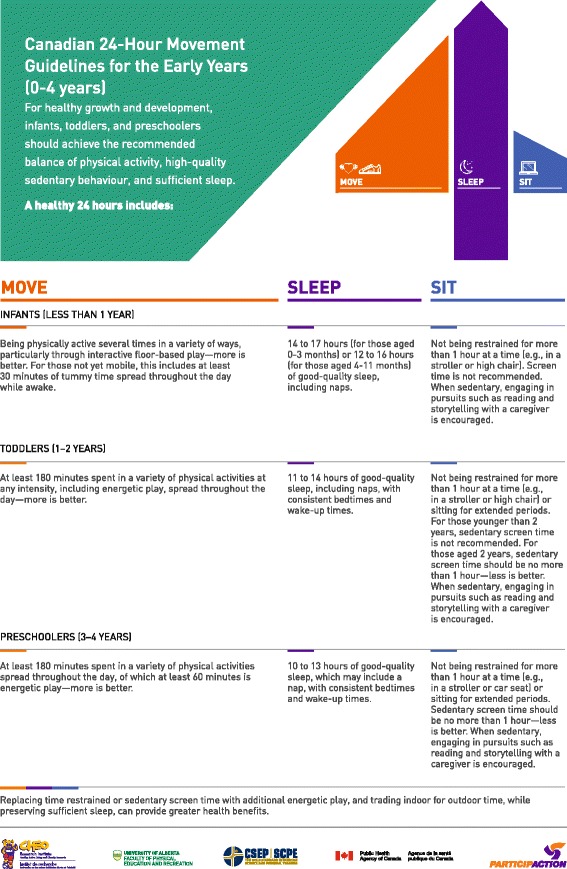
Canadian 24-Hour Movement Guidelines for the Early Years (0–4 years): *An Integration of Physical Activity, Sedentary Behaviour, and Sleep* – English Guidelines. © Canadian Society for Exercise Physiology, 2017. All rights reserved

**Fig. 4:**
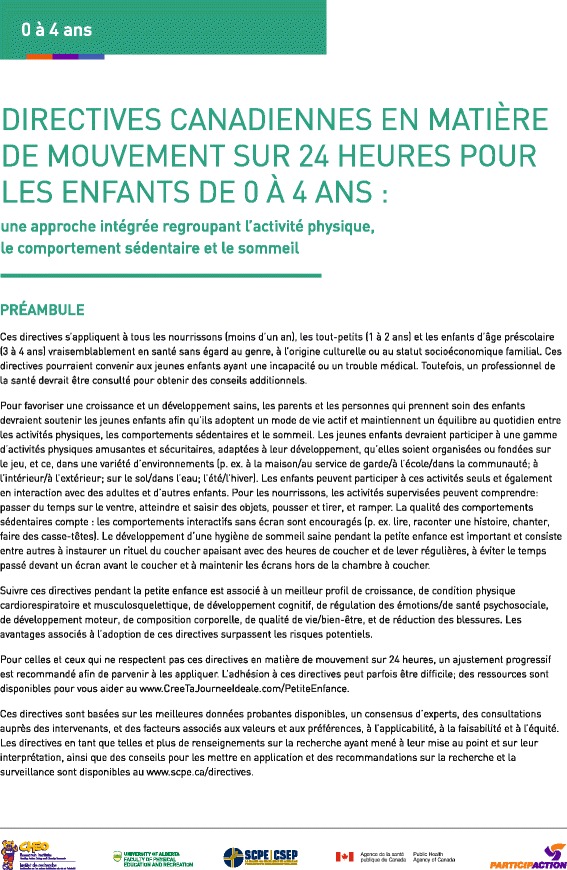
Directives canadiennes en matière de mouvement sur 24 heures pour les enfants de 0 à 4 ans: *une approche intégrée regroupant l’activité physique, le comportement sédentaire et le sommeil* - French Preamble. © Société canadienne de physiologie de l'exercice, 2017. Tous les droits sont réservés

**Fig. 5:**
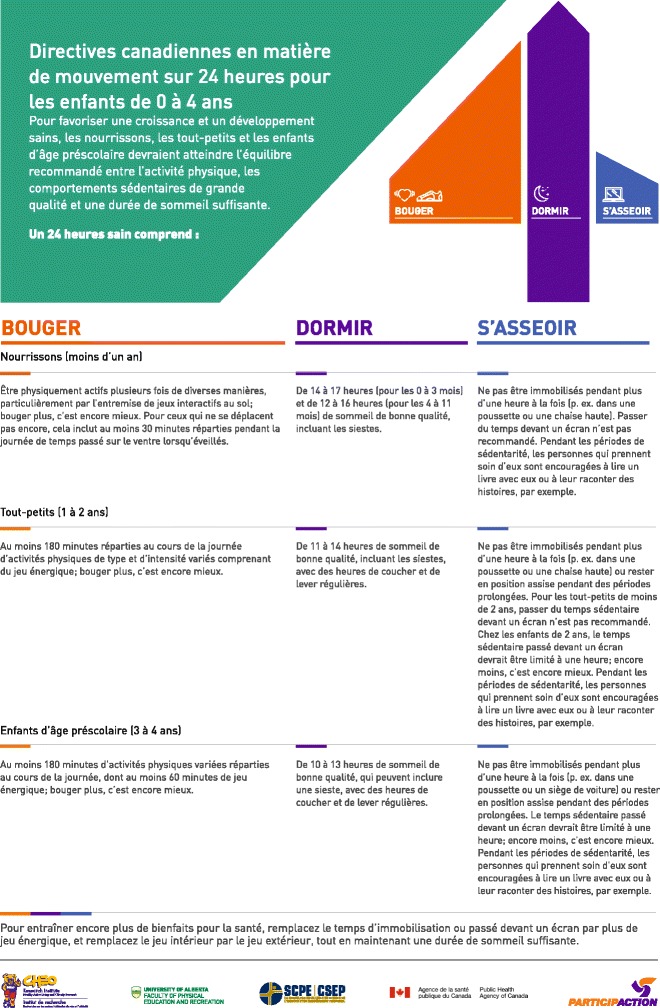
Directives canadiennes en matière de mouvement sur 24 heures pour les enfants de 0 à 4 ans: *une approche intégrée regroupant l’activité physique, le comportement sédentaire et le sommeil* - French Guidelines. © Société canadienne de physiologie de l'exercice, 2017. Tous les droits sont réservés

Three changes that altered the content of the guidelines were made: 1) “while awake” was added to the infant tummy time recommendation to avoid potential harm in having the recommendation misinterpreted as contradicting safe sleep recommendations; 2) based on feedback from the focus groups and from discussions at the Australian guideline development meetings, “energetic play” (i.e., MVPA) was added to the toddler physical activity recommendation; and 3) the term “sedentary” was added to the screen time recommendation based on the Australian Guideline development process and to better reflect the evidence, which did not include data on non-sedentary screen-based activities. Other changes were minor results of the copy-editing process. The final guidelines, with preamble, in English and French are provided in Figs. 2, 3, 4 and 5. The final quality of evidence and strength of recommendation ratings are provided below in the *GRADE Evidence to decision framework: summary* section, including a summary of the rationale for these decisions, with more extensive explanations provided in Additional file 2.

### GRADE evidence to decision framework: Summary

The specific guideline recommendations in the *Canadian 24-Hour Movement Guidelines for the Early Years* are provided below with corresponding statements indicating the quality of the evidence informing the recommendation and the strength of the recommendations.

For infants (less than 1 year), a healthy 24 h includes:

- Being physically active several times in a variety of ways, particularly through interactive floor-based play; more is better. For those not yet mobile, this includes at least 30 min of tummy time spread throughout the day while awake. *Moderate quality evidence, strong recommendation.*
- Not being restrained for more than 1 h at a time (e.g., in a stroller or high chair). Screen time is not recommended. When sedentary, engaging in pursuits such as reading and storytelling with a caregiver is encouraged. *Moderate quality evidence, strong recommendation.*
- 14–17 h (for those aged 0–3 months) or 12–16 h (for those aged 4–11 months) of good-quality sleep, including naps. *High quality evidence, strong recommendation.*

For toddlers (1–2 years), a healthy 24 h includes:

- At least 180 min spent in a variety of physical activities at any intensity, including energetic play, spread throughout the day—more is better. *Moderate quality evidence, strong recommendation.*
- Not being restrained for more than 1 h at a time (e.g., in a stroller or high chair) or sitting for extended periods. For those younger than 2 years, sedentary screen time is not recommended. For those aged 2 years, sedentary screen time should be no more than 1 h; less is better. When sedentary, engaging in pursuits such as reading and storytelling with a caregiver is encouraged. *Moderate quality evidence, strong recommendation.*
- 11–14 h of good-quality sleep, including naps, with consistent bedtimes and wake-up times. *High quality evidence, strong recommendation.*

For preschoolers (3–4 years), a healthy 24 h includes:

- At least 180 min spent in a variety of physical activities spread throughout the day, of which at least 60 min is energetic play—more is better. *Moderate quality evidence, strong recommendation.*
- Not being restrained for more than 1 h at a time (e.g., in a stroller or car seat) or sitting for extended periods. Sedentary screen time should be no more than 1 h; less is better. When sedentary, engaging in pursuits such as reading and storytelling with a caregiver is encouraged. *Moderate quality evidence, strong recommendation.*
- 10–13 h of good-quality sleep, which may include a nap, with consistent bedtimes and wake-up times. *High quality evidence, strong recommendation.*

For all age groups:

- Replacing time restrained or sedentary screen time with additional energetic play, and trading indoor for outdoor time, while preserving sufficient sleep, can provide greater health benefits. *Very low quality evidence, strong recommendation.*

#### Strength of recommendations

The GDP followed the GRADE system to make determinations about the strength of each recommendation by considering; 1) the quality of the supporting evidence, 2) the values and preferences of stakeholders and end-users, 3) whether the recommendations would be considered a wise use of resources, 4) equity, acceptability, and feasibility, and 5) whether the potential benefits outweigh the potential harms [41].

1. *Overall quality of the evidence supporting the recommendations.*
  The quality of the evidence from the four systematic reviews informing the recommendations was considered in the process of assessing the overall quality of the evidence supporting each recommendation [42, 43]. After considering the quality of the evidence associated with the five “critical” health indicators examined in the physical activity systematic review, the GDP did not change the previous physical activity recommendation [26] despite the “low” quality evidence on adiposity and motor development, and the “very low” quality evidence on fitness [18]. The overall certainty of the evidence supporting the recommendation is “moderate”. The panel reached this conclusion based on the fact the “moderate” quality evidence indicating that physical activity improves psychosocial health and cognitive development was considered sufficient to support a recommendation in favour of increasing physical activity [18]. The quality assessment of “moderate” reflects moderate confidence that the true effect is likely to be close to the estimate of the effect presented in the systematic review, but there is a possibility that it is substantially different.
  After considering the quality of the evidence associated with the four “critical” health indicators in the sedentary behaviour review, the GDP did not change the sedentary behaviour recommendation [27]. Despite the “moderate” quality evidence that sedentary behaviour may not affect adiposity and the “very low” quality evidence showing inconclusive findings for a relationship between sedentary behaviour and motor development or cognitive development, there was “moderate” quality evidence that low sedentary behaviour is favourably associated with psychosocial health [21]. This was considered sufficient to support a recommendation in favour of reducing sedentary behaviour. Therefore, the overall certainty of the evidence supporting the recommendation was “moderate”, which reflects moderate confidence that the true effect is likely to be close to the estimate of the effect presented in the systematic review, but there is a possibility that it is substantially different.
  After considering the quality of the evidence associated with the five “critical” health indicators associated with the sleep review, the GDP concluded that despite the “low” quality evidence that sleep duration is not related to adiposity, and the “very low” quality evidence that sleep duration is not related to motor development or growth, “high” quality evidence indicating that longer sleep durations are associated with improved cognitive development and emotional regulation [22] was sufficient to support a recommendation in favour of longer sleep durations. As such, the GDP concluded that the overall certainty of the evidence supporting the recommendation was “high”, which reflects high level of confidence on the part of the GDP that the true effect lies close to the estimate of effect presented in the systematic review.
  After considering the quality of the evidence associated with the six critical health indicators considered in the integrated movement behaviours systematic review [23], the GDP concluded that the overall certainty of the evidence supporting the recommendation was “very low”. The systematic review [23] found “moderate” quality evidence that the most ideal combinations of movement behaviours are not related to growth, “low” quality evidence indicating that the most ideal combinations of integrated behaviours were favourably associated with motor development, and favourably or unrelated to adiposity, and “very low” quality evidence indicating ideal combinations were favourably related to fitness [23]. Furthermore, despite “very low” quality evidence that outdoor time was only related to 1 out of 10 motor skills, “low” quality evidence showed that higher outdoor time was associated with lower sedentary time, and “very low” quality evidence showed that higher outdoor time was associated with lower blood pressure [60]. All six critical health indicators were taken into account in the development of the integrated movement recommendation, including two that were rated as “very low” quality evidence. The assessment of “very low” overall quality of the evidence indicates that the guideline panel has very little confidence in the effect estimate. The true effect is likely to be substantially different from the estimate of effect.
2. *Values and preferences of stakeholders and end-users.*
  While selecting “important” and “critical” health indicators, the GDP considered the importance (i.e., values and preferences) of each indicator to parents, stakeholders and end-users in terms of the development and health of children of the early years. A study was not conducted to have these groups identify important health indicators, per se. However, almost all GDP members were parents, including parents of children of the early years, and several stakeholder and end-user representatives were on the GDP. In addition, many external reviewers of the guidelines were also parents, stakeholders or end-users and provided input during the stakeholder survey; almost all (95.8%) external reviewers indicated the recommendations were important to them. Considering the indirect assessments of target group values and preferences together with the broad range of indicators included in the systematic reviews that informed these recommendations, the GDP concluded that there would likely be no important variability in values and preferences of health indicators if target groups had rated the indicators directly.
3. *Resource requirements (costs).*
  A literature search was conducted using systematic review searching techniques to inform the GDP’s understanding of the expected short-term costs (resource use) required to implement the guidelines as a population-health strategy and to gain insight into the cost-effectiveness of applying these recommendations to children of the in early years. No evidence was found that related to these guidelines. Given the lack of evidence, the GDP sought input from external reviewers (via the stakeholder survey) on their opinions about cost and resource use. Most stakeholders (64.8%) agreed that the costs associated with applying the recommendations would be small or negligible. In terms of the perceived incremental cost relative to the perceived net benefit, most (81.1%) agreed that over the course of a lifetime, the health benefits of applying the recommendations would likely outweigh the costs, which in the judgment of the GDP is likely to generate large savings from a health care systems perspective. In the judgment of the GDP considering the available information, the cost-effectiveness of the recommendations is supported.
4. *Equity, acceptability, and feasibility.*
  A systematic review of the evidence examining equity, acceptability, and feasibility amongst stakeholders was not conducted. Thus, these elements of the recommendations were informed by external reviewer input and by judgments made by the GDP. Most external reviewers (85.5%) agreed that following these recommendations would benefit all groups of the population equally. In the judgment of the GDP, implementing these recommendations would probably increase health equity (i.e., decrease health inequity). Similarly, most external reviewers (78%) indicated that they would “always” or “frequently” use the recommendations. Thus, in the judgment of the GDP, these recommendations are acceptable. Finally, most external reviewers (85%) indicated that in their view the recommendations were “somewhat” to “very easy” to use. Based on this information, in the judgment of the GDP, the recommendations are feasible to implement.
5. *Benefits* vs *harms (justification)*
*Physical Activity:* Based on the systematic review by Carson et al. [18], higher physical activity among children of the early years was favourably related to adiposity, motor development, psychosocial health, cognitive development, and fitness. Although no evidence pointed specifically to harm resulting from increasing physical activity, there was no clear effect observed on bone and skeletal health, cardio-metabolic health, and injuries to rule out the possibility of harm. Nevertheless, in the judgment of the GDP, the benefits of increasing physical activity in children of the early years are likely to outweigh the potential harms, which are likely to be limited to injuries and are unlikely to be serious.
  In balancing the benefits against the harms, in the judgment of the GDP, the desirable indicators (moderate benefits) are likely to outweigh the undesirable indicators (very minor harms); therefore, a recommendation in favour of increasing physical activity is warranted. The GDP placed more value on “moderate” quality evidence showing improvements on adiposity and psychosocial health and on “high” quality evidence showing a benefit in motor development, and less value on “very low” quality evidence showing contradicting findings related to the effect of physical activity on cardio-metabolic health, and on “very low” quality evidence showing that physical activity resulted in an increase in the number of injuries. The GDP also placed more value on evidence showing that total physical activity (TPA), moderate-intensity (MPA), and MVPA resulted in improved fitness.
  A strong recommendation in favour of increasing physical activity is supported by the assessment of overall “moderate” quality evidence supporting the recommendation, the moderate magnitude of the effect, the low variability in how parents and stakeholders value the recommendation, the anticipated small or negligible costs associated with implementing the recommendation, the large savings to the healthcare system expected over the course of a lifetime, and the stakeholder input suggesting that these recommendations would be feasible and acceptable to stakeholders.
*Sedentary behaviour:* According to Poitras et al. [21], limiting sedentary screen-based behaviours was associated with benefits (i.e., psychosocial health and fitness), as was engaging in interactive non screen-based sedentary behaviour with an adult (i.e., storytelling and reading was favourably associated with cognitive development). In terms of evidence of potential harms, there was limited inconclusive “low” and “very low” quality evidence regarding the effects of sedentary behaviour on motor development and injuries. Therefore, the impact of sedentary behaviours on these indicators is uncertain. Nevertheless, in the judgment of the GDP it is highly unlikely that decreasing sedentary time or screen-based behaviours would have an adverse or harmful effect on motor development. The potential harms resulting from limiting sedentary behaviour in children are likely to be minor.
  In considering the balance of the benefits versus the harms, it is the judgment of the GDP that the moderate benefits of limiting/avoiding restrained time, sedentary time, and screen based behaviours outweigh the potential for very minor harms, warranting a recommendation in favour of the sedentary behaviour recommendations. The GDP placed relatively more value on “moderate” quality evidence showing that reducing sedentary time improved adiposity (critical) and psychosocial health (critical), and relatively less value on limited “very low” quality evidence on the impact of sedentary behaviour on motor development (critical) and the lack of evidence about injuries (important).
  The overall quality of evidence supporting the recommendation was deemed to be of “moderate” quality, and the magnitude of the effect is expected to be moderate. Taken together with conclusions drawn from the parent and stakeholder survey, focus groups and interviews (i.e, low variability in how parents and stakeholders value the recommendation, the costs associated with implementing the recommendation are expected to be small or negligible, recommendations would be feasible and acceptable) and the large savings to the healthcare system expected over the course of a lifetime, a strong recommendation is warranted.
  Although the body of evidence indicated that limiting sedentary behaviour is likely to improve health indicators in children in this age group, there was a lack of evidence in relation to the optimal sedentary time in a 24-h day. To address this uncertainty, experts recommended supporting the current *Canadian Sedentary Behaviour Guidelines for the Early Years (aged 0 to 4 years)* [27] from 2012 with slight modifications to introduce guiding principles as to how sedentary behaviours can fit in the context of a healthy day. The recommended sedentary time in the 2012 recommendations align with the new evidence identified by Poitras et al. [21] and, therefore, were adopted by the GDP.
*Sleep:* The systematic review conducted by Chaput et al. [22] showed that longer sleep durations (total sleep in 24 h) was associated with benefits related to emotional regulation, growth, and cognitive development, and reduced sedentary behaviour. With regard to potential harms, there was limited and inconclusive evidence about the impact of sleep on adiposity, motor development, physical activity, injuries and quality of life, and no evidence on cardio-metabolic health. Therefore, the GDP could not be certain of the impact of longer sleep durations on these indicators. However, in the judgment of the GDP, the potential harms resulting from longer sleep durations are likely to be very minor.
  In considering the balance of the benefits versus the harms, it is the judgment of the GDP that the moderate benefits of longer sleep durations are likely to outweigh any potential minor harms, warranting a recommendation in favour of longer sleep durations including napping. The GDP placed more value on “high” quality evidence showing that longer sleep durations improved emotional regulation (critical) and cognitive function (critical), and less value on limited “low” quality and “very low” quality evidence showing that increasing the duration of sleep may impact adiposity or physical activity, and on the lack of evidence examining metabolic health.
  The overall quality of evidence supporting the recommendation was deemed to be of “high” quality and the magnitude of the effect is expected to be moderate; the GDP is very confident that the true effect lies close to the estimate of the effect. Taken together with the conclusions drawn from the parent and stakeholder survey, focus groups and interviews (low variability in how parents and stakeholders value the recommendation, recommendations would be feasible and acceptable, and the anticipated costs associated with implementing the recommendation are expected to be small or negligible), and the large savings to the healthcare system expected over the course of a lifetime, a strong recommendation in favour of longer sleep durations, including napping is warranted.
  Although the body of evidence indicated that longer sleep durations, when compared to shorter sleep durations, were generally favourably associated with health indicators regardless of age, no conclusions could be drawn in terms of optimal durations for infants, toddlers, or preschoolers. Content experts from the GDP pointed to the USA National Sleep Foundation [61] and American Academy of Sleep Medicine [62] guidelines, which recommend that in a 24-h cycle, newborns (0–3 months) sleep 14–17 h, infants (4–11 months) sleep 12–15/16 h, toddlers (1–2 years) sleep 11–14 h, and preschoolers (3–5 years) sleep 10–13 h. This was consistent with the systematic review findings [22], which did not uncover any evidence to warrant deviating from these guidelines. Therefore, the newborn, infant, toddler, and preschooler recommendations were adopted by the GDP.
*Combined movement behaviours:* The systematic review conducted by Kuzik et al. indicated that the most ideal combinations of sedentary behaviour and physical activity were favorably associated with motor development and fitness; both favorably and not associated with adiposity; and not associated with growth [23]. Replacing sedentary time with vigorous physical activity was found to be beneficial for fitness. The most ideal combinations of sleep and sedentary behaviour were favorably associated with adiposity. There was no evidence on harms/injuries. Therefore, it is unclear if there would be an impact of combined movement behaviours on this indicator. However, in the judgement of the GDP, the potential harms resulting from increasing physical activity, decreasing sedentary behaviours, and increasing sleep duration are likely to be very minor.
  In considering the balance of the benefits versus the harms shown in the four systematic reviews, it is the judgement of the GDP that the potential benefits associated with the most ideal combinations of movement behaviours are likely to outweigh any potential minor harms, warranting a strong recommendation in favour of engaging in higher physical activity, less time restrained and less sedentary screen time, and longer sleep durations [18, 21–23]. The GDP also considered previously published evidence that indicated improved health benefits, higher physical activity, and lower sedentary time when children of the early years are outdoors [60, 63]. The GDP placed relatively more value on “very low” quality evidence showing that the most ideal combinations of movement behaviours were associated with adiposity (critical) and fitness (critical), and “very low” quality evidence on motor development (critical), and relatively less value on the limited “very low” quality evidence on growth (critical) and the lack of evidence on psychosocial health/emotional regulation (critical), cognitive development (critical), bone and skeletal health (important), cardiometabolic health (important), and injuries/harms (important).
  The overall quality of evidence supporting the recommendation was deemed to be of “very low” quality and the magnitude of the effect is expected to be very low. Taken together with the conclusions drawn from the parent and stakeholder survey, focus groups and interviews (i.e., low variability in how parents and stakeholders value the recommendation, recommendations would be feasible and acceptable, and the anticipated costs associated with implementing the recommendation are expected to be small or negligible), and the large savings to the healthcare system expected over the course of a lifetime, a strong recommendation is warranted.
  The body of evidence showed that replacing sedentary time or light physical activity with energetic play (MVPA) is likely to improve health indicators in children of the early years. However, there was no information available about combinations of all three movement behaviours included in the systematic review that would inform a specific recommendation for the amounts of sedentary time to be traded for light, moderate, and vigorous physical activity, and sleep.

#### Subgroup considerations

Most stakeholders agreed that if implemented in all Canadian children of early years age, the recommendations would benefit all groups of the Canadian population equally. A few raised concerns about the difficulty that families from low socio-economic status may have in meeting these guidelines. In the judgment of the GDP, these are implementation issues, many of which could be addressed by developing knowledge translation tools targeting families with low socio-economic status. Therefore, the GDP decided not to issue a separate recommendation for this subgroup of the population.

A more detailed version of the “evidence to decision framework” is provided in Additional file 2.

### Dissemination, implementation and evaluation plans

The *Canadian 24-Hour Movement Guidelines for the Early Years (0–4 years): An Integration of Physical Activity, Sedentary Behaviour, and Sleep* were officially released on November 20, 2017 through a comprehensive media relations strategy to optimize exposure and coverage. Dissemination, implementation, communication, and evaluation plans for the new guidelines intend to build on work that is ongoing with the *Canadian 24-Hour Movement Guidelines for Children and Youth* [1], including the development of a comprehensive marketing plan focused on a digital marketing platform, and adaptation of the visual identity and creative concept (“Build your best day” – www.buildyourbestday.com) that will enable clear, consistent and targeted communication with early childhood educators, primary care practitioners, and public health promoters, and indirectly with parents/caregivers. Proactive national media relations outreach, hard copy and e-distribution of guideline-related materials have been orchestrated through Leadership Committee distribution networks. A cross-Canada lecture tour in both French and English is also planned to raise knowledge and awareness of the guidelines among important stakeholders and end-users, including researchers. Webinars targeted to different end-user groups were developed, delivered, and preserved on-line (www.csep.ca/guidelines). All promotional materials, campaigns, and initiatives are available in both English and French.

The visual identity created for the *Canadian 24-Hour Movement Guidelines for Children and Youth* was adapted for use with the *Canadian 24-Hour Movement Guidelines for the Early Years (0–4 years)* (see Figs. 3 and 5) and used to create a digital marketing platform targeted at practitioners who serve children and families of children in the early years. This visual identity provides a consistent, clear, and recognizable look, tone, and feel for the early years guidelines, facilitating increased awareness and recognition of the guidelines.

A digital platform was created as a knowledge portal that provides the three key audiences identified through stakeholder consultation (i.e., early childhood educators, healthcare providers, parents) with the information and resources they need to understand and implement the new guidelines. This resource will be available at www.buildyourbestday.com/earlyyears  by March 31, 2018. The site will provide simple, informational content and resources related to this age group including an instructional video that explains the guidelines, a suite of digitized tools for download, a variety of public-facing and easy-to-read blog articles, infographic(s), poster(s), printable messaging materials (e.g., brochures, tip sheets), and promotional content pieces such as Twitter cards, Facebook images, Instagram images, sample ads, and sample social media content.

Metrics on the success of the guidelines launch will be gathered including traditional media impressions, social media activity, hard-copy and electronic distribution in the first two weeks post-launch as well as the general tone (positive/negative) of any media coverage. Canadian parents’ baseline awareness of the guidelines immediately post launch will be collected via a ParticipACTION survey consistent with evaluation procedures being employed with the guidelines for children and youth. Beliefs among identified key stakeholders (primary care providers, public health promoters, and early childhood educators) about the relative benefits of the 24-h movement guidelines (integrated physical activity, sedentary behaviour, and sleep) versus separate guidelines for each behaviour are being assessed via an online, survey.

An unplanned outcome of the development of these new Canadian guidelines was that they initiated the development of similar guidelines in Australia [59]. Indeed, the Australian guidelines “adoloped” [58] the Canadian guidelines and the concurrent release of both guidelines was coordinated facilitating international collaboration, reducing duplication of effort and enhancing enthusiasm and excitement in both jurisdictions. This bilateral cooperation enticed the World Health Organization to commence the development of *Global 24-Hour Movement Guidelines for the Early Years* (see www.who.int/end-childhood-obesity/news/public-consultation-2017/en for further details). This global momentum should facilitate awareness and uptake of the various jurisdictional guidelines.

### Research gaps

The systematic reviews, GDP meetings, and stakeholder and end-user consultations highlighted several research needs that are listed in Table 3. Briefly, in all three movement behaviour domains (i.e., physical activity, sedentary behaviour, and sleep), more research is required focusing on the dose-response relationships between these behaviours and important health indicators. Few studies to date have used valid and reliable measures of sedentary behaviour or sleep, focused on infants or toddlers, or controlled for important confounders (e.g., diet). The range of health indicators with available data was limited with a lack of evidence for the relationships between the three movement behaviours and fitness, bone and skeletal health, cardiometabolic health, and risk/harms. The change to 24-h movement guidelines has highlighted several additional research gaps. Currently, limited evidence is available on the combined effects of physical activity, sedentary behaviour, and sleep on health in the early years. Future research should focus on examining the combined effect of these behaviours while developing innovative ways to analyze these 24-h data.

**Table 3:** Research gaps identified through the guideline development process

Research needs arising from systematic reviews
• Overall, there is a need for high-quality studies with strong designs (e.g., randomized controlled trials or longitudinal studies, larger sample sizes, objective measures). • To enable comparison across studies, objective measures of sedentary behaviour, physical activity, and sleep (e.g., accelerometry, inclinometry) are needed. Additionally, there is a need to standardize measurement procedures. • To establish the true effect of sedentary behaviour, physical activity, and sleep, possible confounders (e.g., diet) need to be controlled for in studies. • To understand possible dose-response relationships between health outcomes and movement behaviours, examination of the effect of different doses (i.e., duration, frequency) of physical activity, sedentary behaviour, and sleep on health outcomes is needed (e.g., the effect of participating in physical activity for 15 min/day versus 30 min/day versus 60 min/day) and baseline physical activity should be controlled in intervention studies. • Studies in infants and toddlers are required to establish developmentally-appropriate doses of sedentary behaviour and physical activity for these age groups. • Examination of the associations between physical activity and psychosocial health, fitness, bone and skeletal health, cardiometabolic health, and risk/harms are needed. • Examination of the associations between sedentary behaviour and bone and skeletal health, cardiometabolic health, fitness, and risks/harms are needed. • Exploration of the associations between total sedentary time and health outcomes as well as patterns of sedentary behaviour (e.g., combination of timing, length, order of sedentary behaviours relative to physical activity and sleep, and breaks in sedentary behaviours) and health outcomes are needed; • Studies examining the impact of new screen-based devices (e.g., mobile phones, tablets) and other common sedentary behaviours (e.g., reading, puzzles) on health outcomes are needed. • Examination of the associations between sleep and motor development, growth, cardiometabolic health, and risk/harms are needed. • Given the notable differences in development during the early years, studies focusing on sleep should report results based on narrow age ranges (i.e., newborns, infants, toddlers, and preschoolers). • There is a need to determine the distribution of daily movement behaviours for optimal health throughout the early years, more specifically a need for studies that use more balanced approaches to intervene on various movement behaviours in the early years. • Examination of the relationships between combinations of movement behaviours and health indicators is needed.
Research needs arising from Guideline Development Panel meetings and discussions
• Physical Activity
◦ Whether the environment in which physical activity takes place (e.g., indoor vs. outdoor) influences the relationships with health indicators is unclear; using accurate measures to capture physical activity dose together with context is recommended (e.g., combining objective measures of physical activity with time-use diaries). ◦ Explore the differences between types and context (e.g., outdoors, organized, social) of physical activity and their association with health. ◦ The effects of light-intensity physical activity on health indicators in the early years remain unclear. There is need to examine whether activities at the higher end of light physical activity are more beneficial for health than those at the lower end of light physical activity.
• Sedentary Behaviour
◦ Some time spent sedentary may be required to enhance growth and development. The need for a minimum amount of sedentary time to improve growth and development remains to be determined. ◦ There is a need for the use of valid and reliable measures of sedentary behaviour in the early years (e.g., inclinometers). In addition, valid and reliable tools to measure sedentary behaviour in non-ambulatory infants need to be developed. ◦ Establish whether the effect of screens on several health outcomes is due to the use of screens or the lack of movement. ◦ Explore the effects of different types of sedentary behaviour content (e.g., educational vs recreational screen time) on different health indicators.
• Sleep
◦ Research studies focusing on sleep quality are needed (e.g., sleep efficiency, sleep consolidation, sleep architecture). ◦ Identify optimal ranges of sleep duration for the different age groups. Studies examining the effect of different sleep durations on health outcomes are required. ◦ Examine the effect of sleep routines (e.g., consistent bed/wake times, screen time before bed) on sleep quantity and quality.
• Integrated movement behaviours
◦ No cause-effect evidence exists with regard to 24-h movement patterns. Longitudinal and experimental studies are needed. ◦ Exploration of different health indicators (e.g., school readiness) that may be uniquely important during the early years. ◦ Identify additional methods for analyzing 24-h movement data.
Stakeholder, intermediary, and end-user consultation and engagement research needs
• There is a need to understand more completely the language and delivery mediums and methods that minimize end-user feelings of guilt and disengagement and maximize motivation and empowerment to implement and achieve the integrated guidelines. • There is a need to understand the nuances of guideline messaging to effectively and efficiently implement and activate the new guidelines in different end-user groups (e.g., parents, grandparents, child care providers, health care providers, early childhood educators).
International and inter-jurisdictional research needs and opportunities
• The dissemination, activation, implementation, impact, and uptake of the new integrated guidelines in different jurisdictions should be examined and compared. • Intra- and inter-jurisdictional acceptance of the new integrated guidelines approach should be assessed and compared.
Other research needs
• There is a need for cost-effectiveness analyses of interventions aiming to improve movement behaviours during the early years. • There is a need to increase the evidence on movement behaviours and health outcomes in young children with physical or mental diseases or disabilities.

### Surveillance recommendations

The Surveillance Sub-committee met several times through teleconferences to discuss and agree upon the surveillance recommendations for the *Canadian 24-Hour Movement Guidelines for the Early Years*, with due consideration to the existing surveillance structures in Canada (Table 4). It is recommended that in order for a child to be considered to have met these new guidelines, all specific recommendations with a check mark in Table 4 should be met. To meet a specific movement behaviour guideline (e.g., physical activity) each recommendation would need to be met (e.g., for preschoolers meeting the recommendations for both average total physical activity per day of ≥180 min and average MVPA per day of ≥60 min is required). It is recommended that future surveillance work for sedentary behaviour concentrate on the following: 1) distinguish between recreational screen time and non-recreational screen time; 2) incorporate new technology (e.g., tablets, smart phones); and 3) specifically capture the duration of screen time that occurs while in a sitting or lying position. Furthermore, future surveillance efforts should attempt to account for multitasking during sedentary behaviours (e.g., eating while watching television and using social media on a small screen device).

**Table 4:** Surveillance recommendations for the Canadian 24-Hour Movement Guidelines for the Early Years

Movement Behaviour	Specific guideline recommendation for a healthy day	Specific surveillance recommendation	Rationale for specific surveillance recommendation	Recommendation for minimum inclusion in overall guideline surveillance^a^
Age category				
Physical activity				
Infants (aged <1 year)	Being physically active several times in a variety of ways, particularly through interactive floor-based play; more is better	None	Currently there are no available benchmarks, further research is required.	✓ ^b^
	For those not yet mobile, this includes at least 30 min of tummy time spread throughout the day while awake	Average total tummy time per day is ≥30 min while awake^c^	The evidence upon which the guideline is based is predominantly comprised of studies that used average or typical tummy time per day in their analyses. An average allows for some normal day-to-day variability.	✓
Toddlers (aged 1–2 years)	At least 180 min spent in a variety of physical activities at any intensity, including energetic play, spread throughout the day; more is better	Average total physical activity per day is ≥180 min with at least some energetic play (MVPA)^c^	The evidence upon which the guideline is based is predominantly comprised of studies that used average or typical physical activity per day in their analyses. An average allows for some normal day-to-day variability. There are currently no benchmarks for the recommended duration of energetic play in this age group.	✓
Preschoolers (aged 3–4 years)	At least 180 min spent in a variety of physical activities spread throughout the day	Average total physical activity per day is ≥180 minutes^c^	The evidence upon which the guideline is based is predominantly comprised of studies that used average or typical physical activity per day in their analyses.	✓
	of which at least 60 min is energetic play; more is better	Average MVPA per day is ≥60 minutes^c^	An average allows for some normal day-to-day variability.	✓
Sedentary behaviour				
Infants	Screen time is not recommended	A typical day includes no screen time^d^	The evidence upon which the guideline is based is predominantly comprised of studies that used average or typical screen time per day in their analyses.	✓
	Not being restrained for more than 1 h at a time (e.g., in a stroller or high chair)	Time spent restrained is ≤1 h at a time^e^	Empirical evidence substantiating this threshold is lacking though this threshold is aligned with earlier guidelines and has met with stakeholder and end-user acceptance (Tremblay et al., 2012)^f^.	
	When sedentary, engaging in pursuits like reading and storytelling with a caregiver is encouraged	None	Currently there are no available benchmarks, further research is required.	
Toddlers	For those younger than 2 years, sedentary screen time is not recommended	A typical day includes no sedentary screen time^d^	The evidence upon which the guideline is based is predominantly comprised of studies that used average or typical sedentary screen time per day in their analyses.	✓
	For those aged 2 years, sedentary screen time should be no more than 1 h; less is better	Average sedentary screen time per day is ≤1 hour^c^	The evidence upon which the guideline is based is predominantly comprised of studies that used average or typical sedentary screen time per day in their analyses. An average allows for some day-to-day variability in sedentary screen time.	✓
	Not being restrained for more than 1 h at a time (e.g., in a stroller or high chair) or sitting for extended periods	Time spent restrained is ≤1 h at a time^e^	Empirical evidence substantiating this threshold is lacking though this threshold is aligned with earlier guidelines and has met with stakeholder and end-user acceptance (Tremblay et al., 2012)^f^. Currently there are no available benchmarks to be more specific for “sitting for extended periods”, further research is required.	
	When sedentary, engaging in pursuits like reading and storytelling with a caregiver is encouraged	None	Currently there are no available benchmarks, further research is required.	
Preschoolers	Sedentary screen time should be no more than 1 h; less is better	Average sedentary screen time per day is ≤1 hour^c^	The evidence upon which the guideline is based is predominantly comprised of studies that used average or typical sedentary screen time per day in their analyses. An average allows for some day-to-day variability in sedentary screen time.	✓
	Not being restrained for more than 1 hour at a time (e.g., in a stroller or car seat) or sitting for extended periods	Time spent restrained is ≤1 hour at a time^e^	Empirical evidence substantiating this threshold is lacking though this threshold is aligned with earlier guidelines and has met with stakeholder and end-user acceptance (Tremblay et al., 2012)^f^. Currently there are no available benchmarks to be more specific for “sitting for extended periods”, further research is required.	
	When sedentary, engaging in pursuits like reading and storytelling with a caregiver is encouraged	None	Currently there are no available benchmarks, further research is required.	
Sleep				
Infants	14 to 17 h (for those aged 0–3 months) of good quality sleep, including naps	Average total sleep duration per 24 h is 14 to 17 hours^c^	The evidence upon which the guideline is based is predominantly comprised of studies that used average or typical sleep duration per 24 h in their analyses. An average allows for some normal day-to-day variability.	✓
	12 to 16 h (for those aged 4–11 months) of good quality sleep, including naps	Average total sleep duration per 24 h is 12 to 16 hours^c^	The evidence upon which the guideline is based is predominantly comprised of studies that used average or typical sleep duration per 24 h in their analyses. An average allows for some normal day-to-day variability.	✓
Toddlers	11 to 14 h of good quality sleep, including naps	Average total sleep duration per 24 h is 11 to 14 hours^c^	The evidence upon which the guideline is based is predominantly comprised of studies that used average or typical sleep duration per 24 h in their analyses. An average allows for some normal day-to-day variability.	✓
	Consistent bed and wake-up times	Bedtime and wake-up time should not typically vary by more than ±30 min including on weekends^g^	Although the empirical support for a specific surveillance recommendation is weak (Allen et al., 2016)^h^, we propose that sleep schedules (bedtime and wake-up times) should not vary by more than ±30 min each.	
Preschoolers	10 to 13 h of good quality sleep, which may include a nap	Average total sleep duration per 24 h is 10 to 13 hours^c^	The evidence upon which the guideline is based is predominantly comprised of studies that used average or typical sleep duration per 24 h in their analyses. An average allows for some normal day-to-day variability.	✓
	Consistent bed and wake-up times	Bedtime and wake-up time should not typically vary by more than ±30 min including on weekends^g^	Although the empirical support for a specific surveillance recommendation is weak (Allen et al., 2016)^h^, we propose that sleep schedules (bedtime and wake-up times) should not vary by more than ±30 min each.	

*MVPA* moderate- to vigorous-intensity physical activity

^a^The check marks indicate the current recommended minimum inclusion recommendations for surveillance of meeting the 24-h guidelines. Other specific guideline recommendations, which have not been identified as recommended components for surveillance of meeting the 24-h guidelines, should still be measured for descriptive purposes and to determine if changes are occurring prospectively. As evidence grows and surveillance measures evolve for these other recommendations, updates to the minimum surveillance criteria may be required

^b^It is recognized that there is currently no benchmark for this recommendation; however, it remains a recommended component for surveillance of the 24-h guidelines for mobile infants. The implication is that at the present time surveillance of mobile (e.g., crawling or walking) infants meeting the 24-h guidelines is not possible; however, non-mobile infants meeting the tummy time recommendation can be considered to have met the physical activity recommendation and surveillance of meeting the 24-h guidelines for this sub-group is therefore possible

^c^If weekend and weekday measures are available, it is recommended that the average time engaged in each behaviour be weighted 2/7 for weekend days and 5/7 for weekdays to most accurately reflect average weekly measures

^d^It is understood that under special circumstances exposure to screen time may happen but this should be rare or unusual

^e^It is understood that under special circumstances being restrained in excess of 1 h at a time may occur but this should be rare or unusual

^f^Tremblay et al. Canadian Sedentary Behaviour Guidelines for the Early Years (aged 0–4 years). Appl Physiol Nutr Metab 37:370–380, 2012

^g^To accurately assess consistency of bedtime and wake-up time data should be collected on both weekday and weekend days. If data from weekday and weekend days are available, it is recommended that the average variation in bedtime and wake-up time be weighted 2/7 for weekend days and 5/7 for weekdays to most accurately reflect average weekly measures

^h^Allen et al. ABCs of SLEEPING: A review of the evidence behind pediatric sleep practice recommendations. Sleep Med Rev. 29:1–14, 2016

### AGREE II assessment

The four independent assessors scored the procedures used to develop the *Canadian 24-Hour Movement Guidelines for the Early Years (0–4 years): An Integration of Physical Activity, Sedentary Behaviour, and Sleep* following the rubric of the AGREE II [40]. A summary of the combined scores for each AGREE II domain are provided in Table 5. Overall, the guideline development process was scored very high (Domain average ratings 89–100%) and all assessors indicated that they would recommend the guidelines for use. Additional details on all aspects of the guideline development process are available in the Guideline Development Report at www.csep.ca/guidelines.

**Table 5:** Appraisal of Guidelines for Research and Evaluation (AGREE) II reporting grid summary from four independent assessors

AGREE II Item	Reporting Location	Domain Score (%)^b^
Domain 1. Scope and Purpose		100
1. The overall objective(s) of the guideline is (are) specifically described.	• Guideline Development Report • This manuscript	
2. The health question(s) covered by the guideline is (are) specifically described.	• Guideline Development Report • This manuscript • Systematic reviews [18, 21–23] and PROSPERO Registrations	
3. The population (patients, public, etc.) to whom the guideline is meant to apply is specifically described.	• Guideline Development Report • This manuscript	
Domain 2. Stakeholder Involvement		99
4. The guideline development group includes individuals from all the relevant professional groups.	• Guideline Development Report • This manuscript	
5. The views and preferences of the target population (patients, public, etc.) have been sought.	• Guideline Development Report • This manuscript • Focus groups and key informant interviews [33]	
6. The target users of the guideline are clearly defined.	• Guideline Development Report • This manuscript	
Domain 3. Rigour of Development		95
7. Systematic methods were used to search for evidence.	• Guideline Development Report • Systematic reviews [18, 21–23, 60]	
8. The criteria for selecting the evidence are clearly described.	• Guideline Development Report • Systematic reviews [18, 21–23, 60]	
9. The strengths and limitations of the body of evidence are clearly described.	• Guideline Development Report • This manuscript • Systematic reviews [18, 21–23, 60]	
10. The methods for formulating the recommendations are clearly described.	• Guideline Development Report • This manuscript	
11. The health benefits, side effects, and risks have been considered in formulating the recommendations.	• Guideline Development Report • This manuscript • Systematic reviews [18, 21–23, 47, 49, 60]	
12. There is an explicit link between the recommendations and the supporting evidence.	• Guideline Development Report	
13. The guideline has been externally reviewed by experts prior to its publication.	• Guideline Development Report • This manuscript • Focus groups and key informant interviews [33]	
14. A procedure for updating the guideline is provided.	• Guideline Development Report • This manuscript	
Domain 4. Clarity of Presentation		99
15. The recommendations are specific and unambiguous.	• Guideline Development Report • This manuscript	
16. The different options for management of the condition or health issue are clearly presented.^a^	• Not applicable	
17. Key recommendations are easily identifiable.	• Guideline Development Report • This manuscript	
Domain 5. Applicability		89
18. The guideline describes facilitators and barriers to its application.	• Guideline Development Report • This manuscript • Focus groups and key informant interviews [33]	
19. The guideline provides advice and/or tools on how the recommendations can be put into practice.	• Guideline Development Report • This manuscript • CSEP website (www.csep.ca/guidelines)	
20. The potential resource implications of applying the recommendations have been considered.	• Guideline Development Report • This manuscript	
21. The guideline presents monitoring and/or auditing criteria.	• Guideline Development Report • This manuscript	
Domain 6. Editorial Independence		89
22. The views of the funding body have not influenced the content of the guideline.	• Guideline Development Report • This manuscript	
23. Competing interests of guideline development group members have been recorded and addressed.	• This manuscript • Systematic reviews [18, 21–23]	

^a^Item 16 was rated as “not applicable” by one reviewer and assessments from the other reviewers were included in the scaled Domain 4 score

^b^Four independent reviewers applied the AGREE II assessment; the Domain Scores (%) were calculated by summing all the scores of the individual items in a domain and by scaling the total as a percentage of the maximum possible score for that domain (as per the AGREE II Instrument, available at www.agreetrust.org). The “Reporting Location” is not a comprehensive summary of all places where the information in each item can be found. The Guideline Development Report is available at www.csep.ca/guidelines

## Discussion

### Guideline development process and outcomes

This paper outlines the process and outcomes for the development of the *Canadian 24-Hour Movement Guidelines for the Early Years (0–4 years): An Integration of Physical Activity, Sedentary Behaviour, and Sleep*. The development of these guidelines that integrate all movement behaviours follows the shift toward a whole-day approach to conceptualizing movement behaviours that was initiated by the *Canadian 24-Hour Movement Guidelines for Children and Youth: An Integration of Physical Activity, Sedentary Behaviour, and Sleep* [1]. This reconceptualization is supported by existing literature [18, 21–23], compositional analyses from a nationally representative dataset [51], evidence from other behavioural research [64], and logic. As summarized briefly in the results and in more detail in Additional file 2, it is the opinion of the GDP that sufficient evidence exists from all movement behaviour domains to strongly support the final guideline recommendations presented in this paper. The paucity of high quality studies, especially those with experimental dose-response designs, for all movement behaviours is acknowledged and a call for more and better-quality research has been issued.

The guideline development procedures used here followed comprehensive, rigorous, and transparent processes [36, 37, 43], incorporating systematic review findings, consultation findings, and compositional analysis findings, as well as expert, stakeholder, and end-user input. The final guidelines (Figs. 2, 3, 4 and 5) adhere to the structure used with previous Canadian guidelines [1, 26, 27, 65, 66], whereby context is provided for the guidelines through a preamble followed by the guidelines themselves. The preamble and guidelines as presented in Figs. 2, 3, 4 and 5 are intended for practitioners, professionals, stakeholders, and researchers. Additional more user-friendly messaging materials targeted to parents and the general public were developed as outlined in the dissemination, implementation, and evaluation plans section of the Results.

The individual behaviour components of the guidelines have not changed significantly from the earlier *Canadian Physical Activity Guidelines for the Early Years* [26] and *Canadian Sedentary Behaviour Guidelines for the Early Years* [27]. The major change is the integration of all movement behaviours across the 24-h period, with the most notable additions being 1) specific recommendations for sleep duration for all age groups; 2) a recommendation for a duration of 30 min of tummy time spread throughout the day while awake for infants not yet mobile; 3) a recommendation for the inclusion of energetic play in toddlers; 4) a recommendation that preschoolers get at least 60 min of energetic play by age 5 years (changed from the previous recommendation of progression toward at least 60 min of energetic play by age 5 years); and 5) for all age groups, the encouragement of quality sedentary behaviours like reading and storytelling with a caregiver. In addition to providing specific advice for physical activity, sedentary behavior, and sleep, the guidelines also provide some general guidance regarding trade-offs, with the final guideline recommendation being “replacing time restrained or sedentary screen time with additional energetic play, and trading indoor for outdoor time, while preserving sufficient sleep, can provide greater health benefits”. It is also worth highlighting that the GDP used neutral language purposefully, not advocating for or against naps in preschoolers (“which may include a nap”), as it was interpreted that the evidence for health benefits one way or the other was equivocal.

It is possible that busy parents may initially view the 24-h guidelines as just another challenge and potential source of stress. Nevertheless, the overall conceptualization of the integrated approach to all behaviours on the movement continuum holds wide appeal with stakeholders and end-users [1, 15, 16, 33, 67] and they are perceived to be acceptable, affordable, feasible, and realistic.

Agreement existed amongst the GDP that proceeding with the 24-h guidelines for the Early Years using the best available evidence, expert consensus, and stakeholder and end-user input, while being transparent about the quantity and quality of the evidence base and the strength of the guideline recommendations, was the most responsible approach in providing public health recommendations regarding movement behaviours for a healthy day for children of the early years. The GDP believes that the evidence is supportive of all the recommendations, and the potential for benefits still exists even where the evidence is weakest, while the likelihood of harm is very small. Challenges to these recommendations are encouraged and will result in more refined and informed guideline recommendations in the future.

Despite being presented as “24-h movement guidelines”, it is not possible to give precise recommendations that add exactly to 24 h, because there are ranges provided for all behaviour components (e.g., 11–14 h of sleep, at least 60 min of energetic play, no more than 1 h of recreational screen time). Obviously if one child sleeps 11 h and another 14 h, the former has three additional hours of time to be distributed among the wake-time behaviours. Moreover, some degree of day-to-day variability is normal, and provision of ranges allows for this flexibility, accommodating to different schedules and changes in schedules. Collectively, guidance for a healthy 24-h period is provided.

A study examining the proportions of preschool-aged Canadian children (3–4 years) meeting the new guidelines and different recommendations within the guidelines was completed using CHMS data [24]. Associations of meeting the guidelines with adiposity indicators were also explored. Approximately 13% of preschool-aged children met the overall 24-h movement guidelines (as defined by the surveillance recommendations in Table 4), and 3% met none of the three recommendations. Most preschool children met the sleep duration (84%) and physical activity (62%) recommendations, while only 24% met the screen time recommendation. No associations were found between meeting individual or combined recommendations, and anthropometric measures of adiposity. It may be that these anthropometric measures are not sensitive enough to detect differences in adiposity that are actually present. It may be more appropriate to measure other indicators of holistic health in this age group. This is the first study to employ the new guidelines for surveillance assessments in Canada [24]. The findings related to screen time provide evidence for the importance of the recommendations from the recent Canadian Paediatric Society Position Statement that advocates for minimizing, mitigating, mindfully using and modeling healthy use of screens [30].

### Release, dissemination, implementation, and activation planning

A suite of materials to complement and message the guideline recommendations to various subgroups was developed and is publicly available at www.csep.ca/guidelines. These materials present a relative advantage over previous guidelines, in that they show how the guidelines can be easily assimilated into current practice by organizations (compatibility), and how they can most clearly and succinctly be conveyed (simplicity). These clear, supporting messages are essential to informing stakeholders, parents, early childhood educators, public health/health care professionals, and governments, of the value and use of the 24-h movement guidelines. The guidelines themselves provide evidence-informed targets associated with health benefits for children of the early years to follow (under the direction of caregivers).

The Leadership Committee partners (CSEP, HALO, ParticipACTION, University of Alberta, PHAC) will take the lead to disseminate the new guidelines to Canadians both directly and through the partners’ networks. Each partner has a national mandate related to the promotion of healthy active living to Canadians. In addition, the knowledge user representatives on the GDP come from diverse fields concerned with the movement behaviours of children of the early years, and are committed to mobilizing this work to various settings and populations. CSEP will embed these new guidelines into their training materials in order to ensure CSEP members as well as public health professionals, health care practitioners, teachers, and parents understand the importance of all movement behaviours across the 24-h period. As with previous guidelines, ParticipACTION will actively participate in the dissemination, promotion, implementation, and activation of these new guidelines.

In order to motivate adoption, the new guidelines should be followed up with messages that explain the “why” and “how” to the various stakeholder groups, as well as sustained implementation and activation strategies [1, 57, 67]. The suite of prepared messaging and communication tools, adapted visual identity, and digital platform are designed to serve as a foundation for a long-term, multi-platform, multi-sector, multi-jurisdictional, and multi-disciplinary marketing and communication efforts to facilitate uptake and activation of the new guidelines.

The impact and success of the launch of the new guidelines will be assessed through media hits and impressions. Dissemination reach will be assessed with metrics from online and hard-copy distribution. Funding is in place to collect baseline data on parents’ and other stakeholders’ awareness of the new guidelines, allowing for future follow-up research.

Through these various implementation, activation, and evaluation efforts, the long-term goal is that that these guidelines will enhance the promotion of healthy active lifestyles and improved sleep quality and hygiene among infants, toddlers, and preschool children across Canada, and inform healthy active living policy at the local, provincial, and national levels. It is further anticipated that in the long-term this project will provide international leadership and will advance a global healthy active living agenda. Early evidence of success in the form of international leadership can be found with these new Canadian guidelines being the impetus for the development of similar guidelines in Australia [59], New Zealand [68], and the initiation of similar global guidelines by the World Health Organization.

### Updating the guidelines

The final stage in the guideline development cycle is the planning of updates and revisions [36]. It was recommended by the GDP that these guidelines be revisited every 10 years or whenever important new evidence is identified that could inform or alter changes to the existing guideline recommendations. Ten years was recommended as an appropriate period that allows for complete sector penetration and normative utilization by stakeholders, intermediaries, and end-users, while also providing sufficient time for research gaps to be addressed, and is supported by the literature [69, 70].

### Strengths and limitations

There were several strengths of the process used to develop the *Canadian 24-Hour Movement Guidelines for the Early Years (0–4 years): An Integration of Physical Activity, Sedentary Behaviour, and Sleep* including adhering to a rigorous, robust and transparent guideline development process [36, 37, 43]; independent assessment by AGREE II reviewers; involvement of and consultation with a broad assortment of experts, international collaborators, stakeholders, and end-users; consideration of a range of holistic health indicators; using both systematic reviews and novel compositional data analyses to provide a comprehensive evidence base; proactive planning for dissemination, promotion, implementation, and evaluation; and publishing all scholarly work in an open-access, peer-reviewed journal.

Despite these strengths, the guideline development process also had limitations. First, the evidence base for the guidelines was generally incomplete and of low quality, though it does represent the best available evidence collected through systematic reviews and original research. Second, there was very little research available to inform specific aspects of the guidelines (e.g., dose–response studies on behaviour frequency, intensity, duration, type, context). Third, very little research exists on integrated movement behaviours and health indicators in this age group. Because of this limitation, evidence is presently insufficient to provide specific advice on behaviour substitution options for a particular early years child, in a particular situation. Nevertheless, behaviour changes that ensure adequate sleep, reduced screen time, and increased energetic or outdoor play are likely to provide health benefits to most children of the early years. Fourth, it is possible that the various consultation processes used resulted in biased feedback and that voices of important subsets of the population were missed. Fifth, evidence of the cost effectiveness of the guideline recommendations was not available.

### Future research

Specific research needs identified in the development of these guidelines are listed in Table 3. As noted, more research is needed to further inform, substantiate or challenge these new guidelines. Going forward, research should consider the integrated relationships among movement behaviours, and similar integrated 24-h movement guidelines for other age groups (e.g., adults and older adults) should be developed. Such work holds promise in not only creating new opportunities and approaches for healthy lifestyle interventions but also for the discovery of new and novel relationships among movement behaviours, and the underlying physiology and pathophysiology. A need exists for a standardized measurement protocol to collect required information to assess whether the new guidelines are being met. This protocol should be arrived at following a thorough scan of available methods, instruments, and procedures assessed by an expert group, with final consensus recommendations posted and promoted for widespread use.

## Conclusion

The new *Canadian 24-Hour Movement Guidelines for the Early Years (0–4 years): An Integration of Physical Activity, Sedentary Behaviour, and Sleep* are part of a paradigm shift in thinking about daily movement behaviours. This shift from focusing on movement behaviours in isolation to a whole-day approach is supported by the available evidence and stakeholder opinion. These guidelines represent a sensible evolution of public health guidelines whereby optimal health is framed within the balance of movement behaviours across the whole day, while respecting preferences of end-users. The GDP rated these as strong recommendations with the potential benefits of following these guidelines far exceeding the potential risks. It is hoped that these guidelines open new avenues for population health promotion and instigate new research on the health effects of integrated movement behaviours.

## Additional files

Additional file 1: Stakeholder Survey (English followed by French version). (DOC 202 kb)

Additional file 2: Detailed evidence to decision framework explanation for the Canadian 24-Hour Movement Guidelines for the Early Years (0–4 years): An Integration of Physical Activity, Sedentary Behaviour, and Sleep. (DOC 90 kb)

## Abbreviations

AGREE: Appraisal of Guidelines for Research and Evaluation
BMI: Body mass index
CHEO RI: Children’s Hospital of Eastern Ontario Research Institute
CHMS: Canadian Health Measures Survey
CIHR: Canadian Institutes of Health Research
CSEP: Canadian Society for Exercise Physiology
FRCPC: Fellow of the Royal College of Physicians of Canada
GDP: Guideline Development Panel
GRADE: Grading of Recommendations, Assessment, Development and Evaluation
HALO: Healthy Active Living and Obesity research group
LPA: Light-intensity physical activity
MPA: Moderate-intensity physical activity
MVPA: Moderate- to vigorous-intensity physical activity
PA: Physical activity
PHAC: Public Health Agency of Canada
PROSPERO: International Prospective Register of Systematic Reviews
REDCap: Research Electronic Data Capture
SB: Sedentary behaviour
TPA: Total physical activity
WC: Waist circumference
