# Supplementary material for: Canadian 24-Hour Movement Guidelines for the Early Years (0–4 years): An Integration of Physical Activity, Sedentary Behaviour, and Sleep
Source: BMC Public Health. 2017 Nov 20;17(Suppl 5):874. doi: 10.1186/s12889-017-4859-6 (PMC5773896; doi:10.1186/s12889-017-4859-6)
Supplement: Supplementary file 1 — Stakeholder Survey (English followed by French version). (DOC 202 kb) [file 12889_2017_4859_MOESM1_ESM.doc]

**Additional File 1: Stakeholder Survey (English followed by French version).**

**Study title:** Stakeholder Survey for the Canadian 24-Hour Movement Guidelines for the Early Years: An Integration of Physical Activity, Sedentary Behaviour, and Sleep

**Investigator:**

- Dr. Mark Tremblay, Healthy Active Living and Obesity Research Group, Children's Hospital of Eastern Ontario (CHEO), email: [mtremblay@cheo.on.ca](mailto:mtremblay@cheo.on.ca)

As a childcare provider, practitioner or researcher whose work is in some way connected with physical activity, sedentary time and/or sleep, you are being invited to participate in a survey soliciting your opinion on a draft of the *Canadian 24-Hour Movement Guidelines for the Early Years: An Integration of Physical Activity, Sedentary Behaviour, and Sleep* (herein referred to as the*24-Hour Guidelines*). Please note that this is **different** from the survey you may have received last year in relation to new guidelines for children and youth.

With leadership from the Canadian Society for Exercise Physiology, a group of Canadian and International research and practice experts in physical activity, sedentary behaviour, sleep, and health promotion met to develop the *24-Hour Guidelines.* After reviewing and consolidating the evidence, the experts have produced an initial version of the *24-Hour Guidelines*.

One of the final stages in the development of the *24-Hour Guidelines* is to gain feedback about the clarity of the guidelines, as well as level of agreement, perceived importance, applicability, feasibility, resource implications, and equity. Acceptance and dissemination of the *24-Hour Guidelines* is important for the alignment of strategic efforts in policy, practice, and research aimed at promoting health for Canadian infants, toddlers and preschoolers.

*Participation in this survey is voluntary. By accessing and completing this survey you are giving your implied/passive consent to participate in the survey.*  *Your responses will not be linked to your name or email address and responses will be presented in group format only. If you have any questions about this study, please contact Dr. Mark Tremblay at 613-737-7600 ext. 4114 or*[*mtremblay@cheo.on.ca*](mailto:mtremblay@cheo.on.ca)*. The Children's Hospital of Eastern Ontario (CHEO) Research Ethics Board (REB) has reviewed this protocol. The REB considers ethical aspects of all research studies involving human participants at the CHEO and its Research Institute. If you have any questions about your rights as a study participant, you may contact the CHEO REB Chairperson at 613-737-7600 ext. 3624.*

*Note: The “Canadian 24-Hour Movement Guidelines for the Early Years” are in draft form and are not intended for general circulation.*

Thanks for your time!

**We encourage you to circulate the survey link to your colleagues and among your networks. This stakeholder survey will be open until midnight EST on April 9th, 2017.**

**1) Select “Yes” to begin the survey.**

* must provide value

- *Yes*

| Title  Canadian 24-Hour Movement Guidelines for the Early Years: An Integration of Physical Activity, Sedentary Behaviour, and Sleep  2) The Title is clearly stated.   - Strongly Agree - Somewhat Agree - Neither Agree Nor Disagree - Somewhat Disagree - Strongly Disagree   3) Do you agree with the Title?   - Strongly Agree - Somewhat Agree - Neither Agree Nor Disagree - Somewhat Disagree - Strongly Disagree   4) In the box below, please enter comments that you would like to add/change regarding the Title of the Guidelines (above). |
| --- |

Preamble

These guidelines are relevant to all apparently healthy infants (aged < 1 year), toddlers (aged 1-2 years), and preschoolers (aged 3-4 years), irrespective of gender, race, ethnicity, or the socio-economic status of the family. These guidelines may be appropriate for young children with a disability or medical condition; however, a health professional should be consulted for additional guidance. 

For healthy growth and development, parents and caregivers should support young children to live an active lifestyle with a daily balance of physical activities, sedentary behaviours, and sleep. Young children should participate in a range of developmentally appropriate, enjoyable and safe play-based and organized physical activities in a variety of environments (e.g., home/child care/school/community; indoors/outdoors; land/water; summer/winter), by themselves as well as interacting with parents, caregivers, and other children. For infants, supervised activities could include tummy time, reaching and grasping, pushing and pulling, and crawling. The quality of sedentary behaviour matters; for example, interactive non-screen based behaviours are encouraged (e.g., reading, storytelling, singing, puzzles). Developing healthy sleep hygiene in the early years is important, including a calming bedtime routine with consistent bed and wake times, avoiding screen time before sleep, and keeping screens out of the bedroom. 

Following these guidelines through the early years is associated with better growth, cardiorespiratory and musculoskeletal fitness, cognitive development, psychosocial health/emotional regulation, motor development, body composition, and reduced injuries. The benefits of following these guidelines far exceed potential risks.

For those not currently meeting these 24-hour movement guidelines, a progressive adjustment toward them is recommended. Adhering to these guidelines may be challenging at times; resources are available for assistance [insert hyperlinks]. 

These guidelines were informed by the best available evidence, expert consensus, stakeholder consultation, and consideration of values and preferences, applicability, feasibility, resource use (cost) and equity. The specific guidelines and more details on the background research, their interpretation, guidance on how to achieve them, and recommendations for research and surveillance are available at [www.csep.ca/guidelines](http://www.csep.ca/guidelines).

5) The Preamble is clearly stated.

- Strongly Agree
- Somewhat Agree
- Neither Agree Nor Disagree
- Somewhat Disagree
- Strongly Disagree

6) Do you agree with the Preamble?

- Strongly Agree
- Somewhat Agree
- Neither Agree Nor Disagree
- Somewhat Disagree
- Strongly Disagree

7) Would you use (e.g., circulate) the Preamble?

- Always
- Frequently
- Occasionally
- Seldom
- Never

8) In the box below, please enter comments that you would like to add/change regarding the Preamble (above).

***24-Hour Guidelines***

For healthy growth and development, infants, toddlers, and preschoolers should achieve the recommended balance of physical activity, high-quality sedentary behaviour, and sufficient sleep.

**Infants (aged < 1 year)**
For infants, a healthy 24 hours includes:

- Being physically active several times in a variety of ways, particularly through interactive floor-based play; more is better. For those not yet mobile, this includes at least 30 minutes of tummy time spread throughout the day while awake;
- Not being restrained for more than 1 hour at a time (e.g., in a stroller or high chair). Screen time is not recommended. When sedentary, engaging in pursuits like reading and storytelling with a caregiver is encouraged;
- 14 to 17 hours (for those aged 0-3 months) and 12 to 16 hours (for those aged 4-11 months) of good quality sleep, including naps.

**Toddlers (aged 1-2 years)**
For toddlers, a healthy 24 hours includes:

- At least 180 minutes of a variety of physical activities at any intensity spread throughout the day; more is better;
- Not being restrained for more than 1 hour at a time (e.g., in a stroller or high chair) or sitting for extended periods. For those younger than 2 years, screen time is not recommended. For those aged 2 years, screen time should be no more than 1 hour; less is better. When sedentary, engaging in pursuits like reading and storytelling with a caregiver is encouraged;
- 11 to 14 hours of good quality sleep, including naps, with consistent bed and wake-up times.

**Preschoolers (aged 3-4 years)**
For preschoolers, a healthy 24 hours includes:

- At least 180 minutes of a variety of physical activities, of which at least 60 minutes is energetic play, spread throughout the day; more is better;
- Not being restrained for more than 1 hour at a time (e.g., in a stroller or car seat) or sitting for extended periods. Screen time should be no more than 1 hour; less is better. When sedentary, engaging in pursuits like reading and storytelling with a caregiver is encouraged;
- 10 to 13 hours of good quality sleep, which may include a nap, with consistent bed and wake-up times.

Replacing time restrained or in front of a screen with additional energetic play, and trading indoor for outdoor time, while preserving sufficient sleep, can provide greater health benefits.

9) The *24-Hour Guidelines* are clearly stated.

- Strongly Agree
- Somewhat Agree
- Neither Agree Nor Disagree
- Somewhat Disagree
- Strongly Disagree

10) Do you agree with the *24-Hour Guidelines*?

- Strongly Agree
- Somewhat Agree
- Neither Agree Nor Disagree
- Somewhat Disagree
- Strongly Disagree

Using the 24-Hour Guidelines

11) Are the *24-Hour Guidelines* important to you?

- Yes
- No

12) Would you use the *24-Hour Guidelines*?

- Always
- Frequently
- Occasionally
- Seldom
- Never

13) In the box below, please briefly provide any explanation you would like to add for your response in the previous question.

14) How easy or difficult would you find using the *24-Hour Guidelines?*

- Very Easy
- Somewhat Easy
- Neither Easy Nor Difficult
- Somewhat Difficult
- Very Difficult

15) In comparison to separate physical activity, sedentary behaviour and sleep guidelines, do you find these *24-Hour Guidelines…*

- Much More Useful
- More Useful
- Neutral
- Less Useful
- Much Less Useful

16) The costs for you to use, or your organization to implement, the *24-Hour Guidelines* are likely to be small or negligible compared to not using the Guidelines.

- Strongly Agree
- Somewhat Agree
- Neither Agree Nor Disagree
- Somewhat Disagree
- Strongly Disagree
- I Don’t Know
- Not Applicable

17) In the box below, please briefly provide any explanation you would like to add for your response to the previous question.

18) The benefits of using the *24-Hour Guidelines* are likely to outweigh the costs.

- Strongly Agree
- Somewhat Agree
- Neither Agree Nor Disagree
- Somewhat Disagree
- Strongly Disagree
- I Don’t Know

19) In the box below, please briefly provide any explanation you would like to add for your response to the previous question.

20) Following the *24-Hour Guidelines* is likely to benefit all population groups equally, irrespective of gender, race, ethnicity, or the socioeconomic status of the family.

- Strongly Agree
- Somewhat Agree
- Neither Agree Nor Disagree
- Somewhat Disagree
- Strongly Disagree
- I Don’t Know

21) In the box below, please briefly provide any explanation you would like to add for your response to the previous question.

22) Who are the key intermediaries to implement and activate the *24-Hour Guidelines* (e.g., childcare provider, pediatrician, parent)?

23) What supports do these intermediaries need to implement and activate the *24-Hour Guidelines* (e.g., materials, training)?

24) In the box below, please enter any additional comments that you would like to add/change regarding the *24-Hour Guidelines.*

Demographic Questions

25) With what sector do you primarily associate?

- Sport
- Education
- Recreation
- Childcare
- Healthcare
- Public health
- Physical activity/fitness
- Research
- Government
- Other

26) If you selected “other” in the previous question, please explain.

27) Where do you primarily work?

- Alberta
- British Columbia
- Manitoba
- New Brunswick
- Newfoundland and Labrador
- Northwest Territories
- Nova Scotia
- Nunavut
- Ontario
- Prince Edward Island
- Quebec
- Saskatchewan
- Yukon Territory
- Across Canada (i.e., National)
- Outside Canada

28) If you selected “Outside Canada” in the previous question, please identify your country.

29) When the final version of the *24-Hour Guidelines* is complete, would you like to be contacted for final review so that, if supportive, your organization can decide if it would like to be listed in a “supported by” section associated with the *24-Hour Guidelines*?

- Yes
- No
- Don’t Know

30) If you answered “Yes” to the previous question, please provide your email address so that we can send your organization the final version and, if supportive, gather your organization’s name and province/country to be listed in a “supported by” section associated with the *24-Hour Guidelines*.

**Titre du projet :** Sondage destiné aux parties prenantes au sujet des Directives canadiennes en matière de mouvement sur 24 heures pour la petite enfance : une approche intégrée regroupant l’activité physique, le comportement sédentaire et le sommeil

**Chercheur :**

- Mark Tremblay, Ph. D., Groupe de recherche sur les saines habitudes de vie et l’obésité, Centre hospitalier pour enfants de l’est de l'Ontario (CHEO), courriel : [mtremblay@cheo.on.ca](mailto:mtremblay@cheo.on.ca).

En tant qu’intervenant en petite enfance, chercheur ou acteur qui œuvre d’une façon ou d’une autre dans un domaine relié à l’activité physique, à la sédentarité et/ou au sommeil, vous êtes invités à remplir un sondage pour nous faire connaître votre opinion au sujet de la version préliminaire des Directives canadiennes en matière de mouvement sur 24 heures pour la petite enfance : une approche intégrée regroupant l’activité physique, le comportement sédentaire et le sommeil (Directives 24 heures dans le présent texte). Veuillez noter que ce sondage est **différent** de celui que vous avez peut-être reçu l’année dernière au sujet de nouvelles directives pour les enfants et les adolescents.
 
Grâce au leadership de la Société canadienne de physiologie de l’exercice, un groupe d’experts canadiens et internationaux de la recherche et de la pratique sur l’activité physique, le comportement sédentaire, le sommeil et la promotion de la santé a développé ces Directives 24 heures. Après avoir révisé et regroupé les évidences, les experts en ont produit une version préliminaire.
 
L’une des étapes finales du développement de ces Directives 24 heures est de recevoir des commentaires au sujet de la clarté, du niveau d’accord, de l’importance perçue, de l’applicabilité, de la faisabilité, de l’équité et des implications en termes de ressources. L’accueil favorable et la diffusion des Directives 24 heures sont importants pour coordonner les efforts stratégiques pour la création de politiques, la pratique et la recherche ayant pour but la promotion de la santé des nourrissons, des tout-petits et des enfants d’âge préscolaire canadiens.
 
*La participation à ce sondage est volontaire. En accédant à ce sondage et en le remplissant, vous donnez votre consentement implicite/passif à y participer. Nous ne vous demanderons pas votre nom ni votre adresse courriel et les réponses ne seront présentées que sous une forme regroupée. Veuillez communiquer avec Mark Tremblay au 613 737-7600, poste 4114, ou au mtremblay@cheo.on.ca pour toute question au sujet de cette étude. Le comité d’éthique de la recherche (CER) du CHEO a révisé et approuvé ce protocole. Le CER étudie les aspects éthiques de toute recherche qui implique les sujets humains au CHEO et à son Institut de recherche. Si vous avez des questions au sujet de vos droits en tant que participant à l’étude, veuillez communiquer avec le président du CER du CHEO au 613 737-7600, poste 3624.*

Note : Ces Directives canadiennes en matière de mouvement sur 24 heures pour la petite enfance sont présentées en version préliminaire et ne doivent pas être distribuées.
 
Merci pour votre temps!

**Nous vous encourageons à faire circuler le lien de ce sondage à vos collègues et à travers vos réseaux. Le sondage sera ouvert jusqu’à minuit, heure normale de l’Est, le 9 Avril 2017.**

**1) Choisissez « Oui » pour commencer.**

* champ obligatoire

- Oui

Titre

Directives canadiennes en matière de mouvement sur 24 heures pour la petite enfance : une approche intégrée regroupant l’activité physique, le comportement sédentaire et le sommeil

2) Le titre est clairement énoncé.

- Fortement d’accord
- D’accord
- Ni en accord, ni en désaccord
- En désaccord
- Fortement en désaccord

3) Êtes-vous d’accord avec le titre?

- Fortement d’accord
- D’accord
- Ni en accord, ni en désaccord
- En désaccord
- Fortement en désaccord

4) Veuillez inscrire dans la boîte ci-dessous tout commentaire additionnel au sujet de ce que vous voudriez ajouter ou modifier au titre des Directives.

Préambule

Ces directives s'appliquent à tous les nourrissons (âgés de moins d’un an), les tout-petits (âgés de 1 à 2 ans) et les enfants d'âge préscolaire (âgés de 3 à 4 ans) vraisemblablement en santé sans égard au genre, à la race, à l'origine ethnique ou au statut socioéconomique familial. Ces directives pourraient convenir aux jeunes enfants ayant une incapacité ou un trouble médical. Toutefois, un professionnel de la santé devrait être consulté pour obtenir des conseils additionnels.

Pour favoriser une croissance et un développement sains, les parents et les personnes qui prennent soin des enfants devraient soutenir les jeunes enfants afin qu'ils adoptent un mode de vie actif et maintiennent un équilibre au quotidien entre les activités physiques, les comportements sédentaires et le sommeil. Les jeunes enfants devraient participer à une gamme d'activités physiques amusantes et sécuritaires, adaptées à leur développement, qu’elles soient organisées ou fondées sur le jeu, et ce, dans une variété d'environnements (p. ex. à la maison/au service de garde/à l’école/dans la communauté; à l’intérieur/à l’extérieur; sur le sol/dans l’eau; l’été/l’hiver). Les enfants peuvent participer à ces activités seuls et également en interaction avec leurs parents, les personnes qui en prennent soin et d'autres enfants. Pour les nourrissons, les activités supervisées peuvent comprendre : passer du temps sur le ventre, atteindre et saisir des objets, pousser et tirer, et ramper. La qualité des comportements sédentaires compte : les comportements interactifs sans écran sont en effet encouragés (p. ex. lire, raconter une histoire, chanter, faire des casse-têtes). Le développement d'une hygiène de sommeil saine pendant la petite enfance est important et consiste entre autres à instaurer un rituel du coucher apaisant avec des heures de coucher et de lever régulières, à éviter le temps passé devant un écran avant le coucher et à maintenir les écrans hors de la chambre à coucher.

Suivre ces directives pendant la petite enfance est associé à un meilleur profil de croissance, de condition physique cardiorespiratoire et musculosquelettique, de développement cognitif, de régulation des émotions/de santé psychosociale, de développement moteur, de composition corporelle, et de réduction des blessures. Les avantages associés à l’adoption de ces directives surpassent de loin les risques potentiels.

Pour celles et ceux qui ne respectent pas ces directives en matière de mouvement sur 24 heures, un ajustement progressif est recommandé afin de parvenir à les appliquer. L'adhésion à ces directives peut parfois être difficile; des ressources sont disponibles pour vous aider [insérer les hyperliens].

Ces directives sont basées sur les meilleures données probantes disponibles, un consensus d'experts, des consultations auprès des intervenants, et des facteurs associés aux valeurs et aux préférences, à l'applicabilité, à la faisabilité, à l’utilisation des ressources (coûts) et à l'équité. Les directives en tant que telles et plus de renseignements sur la recherche ayant mené à leur mise au point et sur leur interprétation, ainsi que des conseils pour les mettre en application et des recommandations sur la recherche et la surveillance sont disponibles au [www.scpe.ca/directives](http://www.scpe.ca/directives).

5) Le préambule est clairement énoncé.

- Fortement d’accord
- D’accord
- Ni en accord, ni en désaccord
- En désaccord
- Fortement en désaccord

6) Êtes-vous d’accord avec le préambule?

- Fortement d’accord
- D’accord
- Ni en accord, ni en désaccord
- En désaccord
- Fortement en désaccord

7) Utiliseriez-vous le préambule (le distribueriez-vous?)

- Toujours
- Fréquemment
- Occasionnellement
- Rarement
- Jamais

8) Veuillez inscrire dans la boîte ci-dessous tout commentaire additionnel au sujet de ce que vous voudriez ajouter ou modifier au préambule.

Directives 24 heures

Pour favoriser une croissance et un développement sains, les nourrissons, les tout-petits et les enfants d’âge préscolaire devraient atteindre l'équilibre recommandé entre l'activité physique, les comportements sédentaires de grande qualité et une durée de sommeil suffisante.

**Nourrissons (âgés de moins d’un an)**

Pour les nourrissons, un 24 heures sain comprend :

- Être physiquement actifs plusieurs fois de diverses manières, particulièrement par l'entremise de jeux interactifs au sol; bouger plus, c’est encore mieux. Pour ceux qui ne se déplacent pas encore, cela inclut au moins 30 minutes réparties pendant la journée de temps passé sur le ventre lorsqu’éveillés;
- Ne pas être immobilisés pendant plus d'une heure à la fois (p. ex. dans une poussette ou une chaise haute). Passer du temps devant un écran n'est pas recommandé. Pendant les périodes de sédentarité, les personnes qui prennent soin d'eux sont encouragées à lire un livre avec eux ou à leur raconter des histoires, par exemple;
- De 14 à 17 heures (pour les 0 à 3 mois) et de 12 à 16 heures (pour les 4 à 11 mois) de sommeil de bonne qualité, incluant les siestes.

**Tout-petits (âgés de 1 à 2 ans)**

Pour les tout-petits, un 24 heures sain comprend :

- Au moins 180 minutes d'activités physiques variées réparties au cours de la journée, peu importe l'intensité; bouger plus, c'est encore mieux;
- Ne pas être immobilisés pendant plus d'une heure à la fois (p. ex. dans une poussette ou une chaise haute) ou rester en position assise pendant des périodes prolongées. Pour les tout-petits de moins de 2 ans, passer du temps devant un écran n'est pas recommandé. Chez les enfants de 2 ans, le temps passé devant un écran devrait être limité à une heure; encore moins, c’est encore mieux. Pendant les périodes de sédentarité, les personnes qui prennent soin d'eux sont encouragées à lire un livre avec eux ou à leur raconter des histoires, par exemple;
- De 11 à 14 heures de sommeil de bonne qualité, incluant les siestes, avec des heures de coucher et de lever régulières.

**Enfants d'âge préscolaire (âgés de 3 à 4 ans)**

Pour les enfants d'âge préscolaire, un 24 heures sain comprend :

- Au moins 180 minutes d'activités physiques variées réparties au cours de la journée, dont au moins 60 minutes de jeu énergique; bouger plus, c'est encore mieux;
- Ne pas être immobilisés pendant plus d'une heure à la fois (p. ex. dans une poussette ou un siège de voiture) ou rester en position assise pendant des périodes prolongées. Le temps passé devant un écran devrait être limité à une heure; encore moins, c’est encore mieux. Pendant les périodes de sédentarité, les personnes qui prennent soin d'eux sont encouragées à lire un livre avec eux ou à leur raconter des histoires, par exemple;
- De 10 à 13 heures de sommeil de bonne qualité, qui peuvent inclure une sieste, avec des heures de coucher et de lever régulières.

9) Les Directives 24 heures sont clairement énoncées.

- Fortement d’accord
- D’accord
- Ni en accord, ni en désaccord
- En désaccord
- Fortement en désaccord

10) Êtes-vous d’accord avec les Directives 24 heures?

- Fortement d’accord
- D’accord
- Ni en accord, ni en désaccord
- En désaccord
- Fortement en désaccord

11) Les Directives 24 heures sont-elles importantes pour vous?

- Oui
- Non

12) Utiliseriez-vous les Directives 24 heures?

- Toujours
- Fréquemment
- Occasionnellement
- Rarement
- Jamais

13) Veuillez utiliser la boîte ci-dessous pour expliquer brièvement votre réponse à la question précédente.

14) Serait-il facile ou difficile d’utiliser les Directives 24 heures?

- Très facile
- Plutôt facile
- Ni facile, ni difficile
- Plutôt difficile
- Très difficile

15) En comparaison avec des directives séparées sur l’activité physique, le comportement sédentaire et le sommeil, trouvez-vous que ces Directives 24 heures sont…

- Beaucoup plus utiles
- Plus utiles
- Neutre
- Moins utiles
- Beaucoup moins utiles

16) Les coûts engendrés pour vous ou pour votre organisation à la suite de l’utilisation des Directives 24 heures seraient probablement négligeables ou faibles en comparaison à la non-utilisation des Directives.

- Fortement d’accord
- D’accord
- Ni en accord, ni en désaccord
- En désaccord
- Fortement en désaccord
- Je ne sais pas
- Ne s’applique pas

17) Veuillez utiliser la boîte ci-dessous pour expliquer brièvement votre réponse à la question précédente.

18) Les avantages d’utiliser les Directives 24 heures l’emportent probablement sur les coûts.

- Fortement d’accord
- D’accord
- Ni en accord, ni en désaccord
- En désaccord
- Fortement en désaccord
- Je ne sais pas

19) Veuillez utiliser la boîte ci-dessous pour expliquer brièvement votre réponse à la question précédente.

20) Adhérer aux Directives 24 heures est susceptible de présenter des bienfaits égaux à tous les groupes de population, sans égard au genre, à la race, à l’origine ethnique ou au statut socio-économique de la famille.

- Fortement d’accord
- D’accord
- Ni en accord, ni en désaccord
- En désaccord
- Fortement en désaccord
- Je ne sais pas

21) Veuillez utiliser la boîte ci-dessous pour expliquer brièvement votre réponse à la question précédente.

22) Qui sont les principaux intermédiaires dans la mise en œuvre et l’application des Directives 24 heures (fournisseurs de soins en petite enfance, pédiatres, parents)?

23) De quel type de soutien ces intermédiaires auraient-ils besoin pour mettre en oeuvre et appliquer les Directives 24 heures (matériel, formation)?

24) Veuillez inscrire dans la boîte ci-dessous tout commentaire additionnel au sujet de ce que vous voudriez ajouter ou modifier aux Directives 24 heures.

Questions démographiques

25) À quel secteur de pratique êtes-vous principalement associé?

- Sport
- Éducation
- Loisirs
- Soins à l’enfance
- Soins de santé
- Santé publique
- Activité physique/Condition physique
- Recherche
- Gouvernement
- Autre

| 26) Si vous avez choisi « Autre », veuillez préciser : |  |
| --- | --- |

27) Quel est votre principal lieu de travail?

- Alberta
- Colombie-Britannique
- Manitoba
- Nouveau-Brunswick
- Terre-Neuve et Labrador
- Territoires du Nord-Ouest
- Nouvelle-Écosse
- Nunavut
- Ontario
- Île-du-Prince-Édouard
- Québec
- Saskatchewan
- Yukon
- À travers le Canada
- Hors Canada

| 28) Si vous avez choisi « Hors Canada » à la question précédente, veuillez préciser le pays : |  |
| --- | --- |

29) Lorsque la version finale des Directives 24 heures sera prête, aimeriez-vous que l’on communique avec vous pour révision finale et, dans le cas où vous souhaiteriez appuyer les Directives, votre organisation pourrait alors décider si elle souhaite apparaître dans une section « appuis » associée aux Directives 24 heures?

- Oui
- Non
- Je ne sais pas

30) Si vous avez répondu « Oui » à la question précédente, veuillez inscrire votre adresse courriel afin que nous puissions vous envoyer la version finale. Si vous appuyez toujours les Directives 24 heures, nous recueillerons le nom de votre organisation et votre province/pays. Ces renseignements seront placés dans la section « appuis » associée aux Directives.
