# Supplementary material for: Canadian 24-Hour Movement Guidelines for the Early Years (0–4 years): An Integration of Physical Activity, Sedentary Behaviour, and Sleep
Source: BMC Public Health. 2017 Nov 20;17(Suppl 5):874. doi: 10.1186/s12889-017-4859-6 (PMC5773896; doi:10.1186/s12889-017-4859-6)
Supplement: Supplementary file 2 — Detailed evidence to decision framework explanation for the Canadian 24-Hour Movement Guidelines for the Early Years (0–4 years): An Integration of Physical Activity, Sedentary Behaviour, and Sleep. (DOC 90 kb) [file 12889_2017_4859_MOESM2_ESM.doc]

**Additional File 2: Detailed evidence to decision framework explanation for the Canadian 24-Hour Movement Guidelines for the Early Years (0-4 years): An Integration of Physical Activity, Sedentary Behaviour, and Sleep**

Evidence Summary

*Physical activity*

“Low” quality evidence from 1 randomized controlled trial (RCT) showed that recommendations from a nurse to conduct physical activity resulted in a reduction in adiposity (critical indicator) as measured by skinfolds [1]. Four clustered RCTs examined the effect of physical activity on adiposity (critical indicator) and were rated as low quality evidence [2-5]. Only 1 [2] out of the 4 studies showed a decrease in adiposity (as measured by BMI); however, the 3 other studies that showed no improvement also did not show any significant differences in physical activity between the intervention and the control groups, which may explain the no effect observed. Four RCTs examined the impact of physical activity on motor development (critical), which were rated as “low” quality evidence [1, 6-8]. Out of the 4 studies, 3 showed an increase in motor development as measured by total motor development scores [6-8]. In the remaining RCT, there were no differences between the intervention and the control group [1]; however, there were also no significant changes in physical activity, which may explain the lack of effect. “Low” quality evidence from 2 clustered RCTs showed opposing results: in one study physical activity resulted in improvements in motor development in the intervention group (measured by total motor development and jumping) [3], while the second study showed no improvements; however, physical activity was also not significantly different between he the two groups, which could explain the lack of effect [5]. Two RCTs rated as moderate quality evidence showed that planned physical activity resulted in an improvement in psychosocial health (critical), however, it is unknown whether the intervention resulted in changes in physical activity because it was not measured [6, 9]. Two RCTs rated as moderate quality evidence showed that physical activity resulted in improvements in cognitive development (critical), as measured by language development and psychomotor skills [6,7]. However, changes in physical activity between the two groups (intervention and control) were not measured. “Very low” quality evidence from 3 observational studies showed that physical activity may result in improvements in fitness (critical) [10-12]. More specifically, total physical activity (TPA), moderate-intensity (MPA), and moderate to vigorous intensity physical activity (MVPA) showed improvements in cardiorespiratory fitness, muscular fitness, and speed agility.

Low quality evidence from one RCT showed that physical activity did not have an effect on bone and skeletal health (important); however, there was also no difference in physical activity between the intervention and the control, which may explain the lack of effect [13]. “Very low” quality evidence from nine observational studies (eight unique samples) found inconsistent and contradicting findings related to the effect of physical activity on cardio-metabolic health (important) [11, 13-19]. One observational study with evidence rated as “very low quality” concluded that high physical activity level (compared to low activity level) resulted in an increase in number of injuries (important) but not in an increase in risk severity [20].

*Sedentary behaviour*

“Moderate quality” evidence from one RCT showed that decreasing sedentary behaviour (i.e., screen time) may not have an impact on adiposity (critical) at 9-month follow-up as measured by BMI z-scores [21]. One RCT rated as having “moderate” quality evidence showed that a reduction in screen time improved psychosocial health (critical) (i.e., reduced aggressive and delinquent behaviours at 9-months post-intervention) [21. Twenty-five observational studies rated as providing “very low” quality evidence showed inconsistent findings in terms of the absolute effect of screen time on cognitive development (critical) [22-46]; however, when the data were analyzed by type of sedentary behaviour, story-telling and reading showed an improvement in cognitive development, while mobile phone use and screen time resulted in a potential negative impact on cognitive development [46]. “Very low” quality evidence from a single observational study showed that sedentary time did not have an effect on bone and skeletal health (important) as measured by the bone stiffness index [47]. Only one observational study rated as having “very low” quality evidence examined the effect of sedentary behaviour on cardio-metabolic health (important), which showed that television time did not result in high blood pressure [48]. Evidence from two observational studies, rated as “very low” quality, showed that increases in television time was associated with a decrease in fitness (important) as measured by standing long jump performance and physical fitness level [49, 50].

Seven observational studies rated as “very low” quality evidence showed inconsistent findings related to the effect of sedentary behaviour on motor development (critical) [50-56]. No studies in the sedentary behaviour systematic review [57] included evidence on the effect of sedentary behaviour on injuries.

*Sleep*

“High” quality evidence from 2 randomized cross-over trials showed that routine sleep (vs. sleep restriction) improved emotional regulation (critical) meaning that children showed better self-regulation strategies and emotional responses [58, 59]. “High” quality evidence from 1 RCT showed that napping (vs. sleep restriction) was associated with better cognitive function (critical) as measured by correct answers in an explicit recognition task [60]. “Very low” quality evidence from 4 observational studies showed that shorter sleep duration was associated with more sedentary behaviour (important) as measured by screen time [61-64], while 1 other observational study showed consistent findings indicating that longer sleep duration resulted in less screen time [65]. “Very low” quality evidence from 1 observational study showed that longer sleep duration was associated with growth (critical) in infant length in children regardless of gender [66], while 1 other observational study showed the opposite; shorter sleep duration was associated with higher weight-for-length but in girls only [67].

“Low” quality evidence from 26 observational studies showed conflicting results about the impact of sleep duration on adiposity; 18 (10 longitudinal, 8 cross sectional) showed that shorter sleep duration was associated with higher adiposity (critical) [61, 65, 68-83], 7 (2 longitudinal analyses, 6 cross-sectional analyses) showed sleep duration was not associated with adiposity [74, 75, 84-88], and 2 presented opposing results indicating that longer sleep duration was associated with adiposity [66, 89]. “Low” quality evidence from 2 observational studies found that there was no impact of sleep duration on motor development (critical) [86, 90]. “Low” quality evidence from 3 observational studies, showed inconsistent findings about the impact of sleep duration on injuries (important); 2 showed that shorter sleep duration was associated with more injuries [91, 92], while 1 showed sleep duration had no effect on the risk for injuries [93]. “Low” quality evidence from 3 observational studies showed inconclusive findings related to the impact of sleep duration on physical activity (important) (current and future physical activity behaviour) [61, 77, 94]. “Very low” quality evidence from 1 observational study found that shorter sleep duration in the early years (3 years of age) did not have an effect on quality of life (important) later on in life (at 13 years) [95]. No studies in the systematic review included evidence for the impact of sleep duration on cardio-metabolic health (important) [96].

*Integrated behaviours in a 24-hour period*

Two cluster RCTs rated as having “low” quality evidence indicated that increasing physical activity while reducing sedentary behaviour may not reduce adiposity, measured as changes in weight for height indices [97,98], but may reduce body fat [98]. Two cluster RCTs assessed as providing “low” quality evidence showed that reducing sedentary behaviour, while increasing physical activity may improve motor skills [97, 99]. One cluster RCT assessed as “moderate” quality evidence, indicated that reducing sedentary behaviour and increasing physical activity, did not result in changes in growth (height or weight) [98]. “Very low” quality evidence from a single observational study showed that replacing sedentary time with light and moderate intensity physical activity was not favourably associated with fitness [100]. However, replacing sedentary time with vigorous intensity physical activity resulted in improved fitness as measured by 20 m shuttle, standing long jump, and 4X10 m shuttle but not with handgrip strength.

The Guideline Development Panel (GDP) also commissioned a model examining the cross-sectional associations between sleep duration, sedentary time, physical activity and adiposity indicators among Canadian preschool children using compositional analyses [101]. The model showed that the overall composition of movement behaviours was associated with BMI z-scores but not with waist circumference. The quality of the evidence produced by the model was not rated using GRADE.

Overall Quality of the Evidence

*Physical activity*

The GDP identified five indicators as critical for decision-making when developing the recommendations on physical activity: 1) adiposity, 2) psychosocial health, 3) cognitive development, 4) motor development, and 5) fitness. The quality of the evidence for these indicators was rated as follows: 1) “low” quality evidence on adiposity, 2) “moderate” quality evidence on psychosocial health, 3) “moderate” quality evidence on cognitive development, 4) “low” quality evidence on motor development, and 5) “very low” quality evidence on fitness.

After reviewing the evidence, the GDP concluded that the recommendation would remain unchanged despite the “low” quality evidence on adiposity and motor development and the “very low” quality evidence on fitness. In other words, the “moderate” quality evidence demonstrating that physical activity improves psychosocial health and cognitive development is sufficient to support a recommendation in favour of increasing physical activity. Therefore, the GDP concluded that the overall certainty of the evidence supporting the recommendation is “moderate”. The GDP is moderately confident in the effect estimate presented in this body of evidence. The true effect is likely to be close to the estimate of the effect, but there is a possibility that it is substantially different.

*Sedentary behaviour*

The GDP identified 4 indicators as critical for decision-making when developing the recommendations on sedentary behaviour: 1) adiposity, 2) motor development, 3) psychosocial health, and 4) cognitive development. The quality of the evidence for these indicators was rated as follows: 1)” moderate” quality evidence on adiposity, 2) “very low” quality evidence on motor development, 3) “moderate” quality evidence on psychosocial health, and 4) “very low” quality evidence on cognitive development.

After reviewing the evidence, the GDP concluded that the recommendation would remain unchanged despite the “very low” quality evidence on motor development and cognitive development. In other words, the “moderate” quality evidence demonstrating that although reducing sedentary behaviour may not have an impact on adiposity, it is likely to improve psychosocial health, is sufficient to support a recommendation in favour of reducing sedentary behaviour, especially screen time. Therefore, the GDP concluded that the overall certainty of the evidence supporting the recommendation is “moderate”. The GDP is moderately confident in the effect estimate presented in this body of evidence. The true effect is likely to be close to the estimate of the effect, but there is a possibility that it is substantially different.

*Sleep*

The GDP identified 5 indicators as critical for decision-making when developing the recommendations on sleep: 1) adiposity, 2) emotional regulation 3) cognitive development, 4) motor development, and 5) growth. The quality of the evidence for these indicators was rated as follows: 1) “low” quality evidence on adiposity, 2) “high” quality evidence on emotional regulation, 3) “high” quality evidence on cognitive development, 4) “low” quality evidence on motor development, and 5) “very low” quality evidence on growth.

After reviewing the evidence, the GDP concluded that the recommendation and its strength would remain unchanged despite the “low” quality evidence on adiposity and motor development and “very low” quality evidence on growth. In other words, the “high” quality evidence demonstrating that sleep improves cognitive development and emotional regulation is sufficient to support a recommendation in favour of longer sleep durations. Therefore, the GDP concluded that the overall certainty of the evidence supporting the recommendation is “high”. The GDP is very confident that the true effect lies close to the estimate of the effect presented in this body of evidence.

*Parental preferences and values*

Users of the guidelines *per se* did not rate the importance of the indicators included in the review. The rating of indicators was done by the GDP considering what in their view the most important indicators were for end users in deciding physical activity, sedentary behaviour and sleep durations in children. Almost all GDP members were also parents and end users. In addition, many parents provided input in the external review process of the guidelines. Almost all (95.8%) external reviewers consulted (which included many parents) indicated the recommendations were important to them. Considering the broad range of indicators included in the systematic review that informed these recommendations, the GDP concluded that a sample of parents (other than themselves) only would similarly value the main indicators.

*Resource requirements (costs)*

A systematic review of the evidence on cost and resource use related to these recommendations was conducted; however, no evidence was found related to the content of the guidelines. The review found no evidence on the expected short-term resource use required to rollout the recommendation as a population-health strategy or evidence examining the cost-effectiveness of applying these recommendations in the early years. Given the lack of evidence, the GDP sought input from external reviewers on their opinions about cost and resource use. Most stakeholders (64.8%) agreed that the costs associated with applying the recommendations would be small or negligible. In terms of the perceived incremental cost relative to the perceived net benefit, most (81.1%) agreed that over the course of a lifetime, the health benefits of applying the recommendations would likely outweigh the costs, which in the judgment of the GDP is likely to generate large savings from a health systems perspective. Recent work done in Australia assessing the cost-effectiveness of these recommendations also supports this judgment (unpublished analyses available upon request).

Although the review found no studies examining cost-effectiveness, in the judgment of the GDP, and considering input from the stakeholder survey, the cost-effectiveness of the intervention probably favours the intervention (i.e., recommending in favour of increasing physical activity, reducing sedentary behaviour especially screen time and increasing sleep duration).

*Equity, acceptability, and feasibility*

A systematic review of the evidence examining equity, acceptability, and feasibility amongst stakeholders was not conducted. Thus, these elements of the recommendations were informed by stakeholder input and by judgments made by the GDP.

Most external reviewers (stakeholders) (85.5%) agreed that following these recommendations would benefit all groups of the population equally. In the judgment of the GDP, the use of these recommendations would therefore probably increase health equity (i.e., decrease health inequity). Similarly, most external reviewers (78%) indicated that they would "always" or "frequently" use the recommendations. Thus, in the judgment of the GDP, these recommendations are acceptable. Finally, most external reviewers (85%) indicated that in their view the recommendations were "somewhat" to "very easy" to use. Based on this information, in the judgment of the GDP, the recommendations are feasible to implement.

Justification

*Physical activity*

In terms of benefits, the body of evidence [102] showed that in this age group (0 to 4 years of age) increases in physical activity resulted in improvements on adiposity, motor development, psychosocial health, cognitive development, and fitness.

In relation to potential harm, although no evidence pointed specifically to harm resulting from increasing physical activity, no clear effect was observed on bone and skeletal health, cardio-metabolic health, and injuries. Nevertheless, in the judgment of the GDP the potential harms resulting from increasing physical activity in children are likely to be limited to injuries and likely to be very minor.

In balancing the benefits against the harms, in the judgment of the GDP, the desirable indicators (moderate benefits) are likely to outweigh the undesirable indicators (very minor harms); therefore, a recommendation in favour of increasing physical activity is warranted. The GDP placed more value on “moderate” quality evidence showing improvements on adiposity and psychosocial health and on “high” quality evidence showing a benefit on motor development, and less value on “very low” quality evidence showing contradicting findings related to the effect of physical activity on cardio-metabolic health, and on “very low” quality evidence showing that physical activity resulted in an increase in number of injuries. The GDP also placed more value on evidence showing that TPA, MPA, and MVPA resulted in improved fitness.

A strong recommendation in favour of increasing physical activity is supported by the assessment of overall “moderate” quality evidence supporting the recommendation, the moderate magnitude of the effect, the low variability in how parents and stakeholders value the recommendation, the anticipated small or negligible costs associated with implementing the recommendation, the large savings to the healthcare system expected over the course of a lifetime, and the stakeholder input suggesting that these recommendations would be feasible and acceptable to end users.

*Sedentary behaviour*

In terms of potential benefits, the body of evidence [57] showed that in this age group (0 to 4 years of age), reducing sedentary behaviour (especially screen time), was associated with some improved measures of adiposity, psychosocial health, cognitive development (except for story-telling and reading), and motor development.

In relation to harms, there is limited inconclusive “low” and “very low” quality evidence about the impact of sedentary behaviour on motor development and injuries. Therefore, the GDP was unable to say with certitude what the impact of sedentary behaviours will be on these indicators. Nevertheless, in the judgment of the GDP it is highly unlikely that decreasing sedentary behaviours or screen time would have an adverse or harmful effect on motor development. The potential harms resulting from limiting sedentary behaviour in children are likely to be limited to injuries and are likely to be very minor.

In balancing the benefits against the harms, in the judgment of the GDP, the desirable indicators (moderate benefits) are likely to outweigh the undesirable indicators (very minor harms); therefore, a recommendation in favour of limiting sedentary time is warranted. The GDP placed relatively more value on “moderate” quality evidence showing that reducing sedentary time improved adiposity and psychosocial health, which were both critical indicators, and relatively less value on limited conflicting data on the impact of sedentary behaviour on motor development and the lack of evidence about injuries.

Overall the quality of evidence supporting the recommendation was deemed to be of "moderate" quality and the magnitude of the effect is expected to be moderate. The conclusions drawn from the stakeholder survey indicating that there is low variability in how parents and stakeholders value the recommendation, the anticipated small or negligible costs associated with implementing the recommendation, the large savings to the healthcare system expected over the course of a lifetime, and the stakeholder input suggesting that these recommendations would be feasible and acceptable to stakeholders, support a strong recommendation in favour of limiting sedentary time and especially screen time.

Although the body of evidence indicated that limiting sedentary behaviour is likely to improve health indicators in children in this age group, there was a lack of evidence in relation to the optimal sedentary time in a 24-hour day. To address this uncertainty, experts recommended supporting the current *Canadian Sedentary Behaviour Guidelines for the Early Years (aged 0 to 4 years)* from 2012 with slight modifications to introduce guiding principles as to how sedentary behaviours can fit in the context of a healthy day [103]. The recommended sedentary time in the 2012 recommendations align with the new evidence identified by Poitras et al. [57] and therefore, were adopted by the GDP.

*Sleep*

In terms of benefits, the body of evidence showed that in this age group (0 to 4 years of age) longer sleep durations (total sleep in 24 hours) produced benefits related to emotional regulation, growth, and cognitive development, while reducing sedentary behaviour [96].

With regard to potential harms, there is limited and inconclusive evidence about the impact of sleep on adiposity, motor development, physical activity, injuries and quality of life. Also, the review found no evidence examining the effect of sleep on cardio-metabolic health. Therefore, the GDP is unable to say with certitude what the impact of longer sleep durations will be on these indicators. However, in the judgment of the GDP the potential harms resulting from longer sleep durations are likely to be very minor.

In balancing the benefits against the harms, in the judgment of the GDP, the desirable indicators (moderate benefits) are likely to outweigh the undesirable indicators (very minor harms); therefore, a recommendation in favour of longer total daily sleep duration (including naps) is warranted. The GDP placed more value on “high” quality evidence showing that longer sleep durations improved emotional regulation and cognitive function, which were both critical indicators, and less value on limited conflicting “low” quality and “very low” quality evidence showing that increasing the duration of sleep may increase or decrease adiposity or physical activity, and on the lack of evidence examining metabolic health.

Overall, the quality of evidence supporting the recommendation was deemed to be of "high" quality and the magnitude of the effect is expected to be moderate. The overall quality assessment indicating the GDP is very confident that the true effect lies close to the estimate of the effect, the conclusions drawn from the parent and stakeholder survey indicating that there is low variability in how parents and stakeholders value the recommendation, the anticipated small or negligible costs associated with implementing the recommendation and the large savings to the healthcare system expected over the course of a lifetime, and the stakeholder input suggesting that these recommendations would be feasible and acceptable to stakeholders, support a strong recommendation in favour of longer sleep durations (including naps).

Although the body of evidence found that longer sleep durations, when compared to shorter sleep durations, were generally associated with improved health indicators regardless of age, the sleep durations and age groups in the different studies were not consistent and no conclusions could be drawn in terms of optimal durations. Therefore, uncertainty remains regarding the optimal sleep duration ranges that would produce the best health indicators in each age group. Experts pointed to the USA National Sleep Foundation guidelines [104] and the American Academy of Sleep Medicine [105], which recommends that newborns (0-3 months) obtain 14-17 h of sleep, infants (4-11 months) obtain 12-15/16 h of sleep, toddlers (1-2 years) obtain 11-14 h of sleep, and preschoolers (3-5 years) obtain 10-13 h of sleep per 24-hour cycle. Findings of studies included in the systematic review are consistent with current sleep duration recommendations and do not suggest that they should be changed, therefore, those sleep durations were adopted by the GDP [96].

*Subgroup considerations*

Although most stakeholders agreed that the recommendations would benefit all groups of the Canadian population equally, a few raised concerns about the difficulty that families from low socio-economic status may have in meeting these guidelines. In the judgment of the GDP, these are minor implementation issues that could be addressed by developing knowledge translation tools targeting families from low socio-economic status. Therefore, the GDP decided not to issue a separate recommendation for this subgroup of the population.

*Implementation considerations*

Replacing time restrained or sedentary screen time with additional energetic play, and trading indoor for outdoor time, while preserving sufficient sleep, can provide greater health benefits. Although the body of evidence indicated that limiting sedentary behaviour is likely to improve health indicators in children in this age group, there was lack of evidence in relation to the optimal sedentary time in a 24-hour day. To address this uncertainty, experts recommended supporting the current *Canadian Sedentary Behaviour Guidelines for the Early Years (aged 0 to 4 years)* from 2012 [103] with slight modifications to introduce guiding principles as to how sedentary behaviours can fit in the context of a healthy day. The recommended sedentary time in the 2012 recommendations align with the new evidence identified by Poitras et al. [57] and therefore, were adopted by the GDP.

**References**

1. de Vries A, Huiting H, Heuvel E, L'Abée C, Corpeleijn E, Stolk R. An activity stimulation programme during a childs first year reduces some indicators of adiposity at the age of two‐and‐a‐half. Acta Paediatr. 2015;104(4):414-21.

2. Annesi JJ, Smith AE, Tennant GA. Effects of a cognitive–behaviorally based physical activity treatment for 4- and 5-year-old children attending US preschools. Int J Behav Med. 2013;20(4):562-6.

3. Jones RA, Riethmuller A, Hesketh K, Trezise J, Batterham M, Okely AD. Promoting fundamental movement skill development and physical activity in early childhood settings: A cluster randomized controlled trial. Pediatr Exerc Sci. 2011;23(4):600-15.

4. Mo-suwan L, Pongprapai S, Junjana C, Puetpaiboon A. Effects of a controlled trial of a school-based exercise program on the obesity indexes of preschool children. Am J Clin Nutr. 1998;68(5):1006-11.

5. Bonvin A, Barral J, Kakebeeke TH, Kriemler S, Longchamp A, Schindler C, et al. Effect of a governmentally-led physical activity program on motor skills in young children attending child care centers: A cluster randomized controlled trial. Int J Behav Nutr Phys Act. 2013;10:90.

6. Porter LS. The impact of physical-physiological activity on infants' growth and development. Nursing Res. 1972;21(3):210-9.

7. Teixeira Costa HJ, Abelairas-Gomez C, Arufe-Giráldez V, Pazos-Couto JM, Barcala-Furelos R. Influence of a physical education plan on psychomotor development profiles of preschool children. J Human Sport Exerc. 2015;10(1):126-40.

8. Mostafavi R, Ziaee V, Akbari H, Haji-Hosseini S. The effects of Spark physical education program on fundamental motor skills in 4-6 year-old children. Iran J Pediatr. 2014;23(2): 216-9.

9. Lobo YB, Winsler A. The effects of a creative dance and movement program on the social competence of Head Start preschoolers. Soc Dev. 2006;15(3):501-19.

10. DuRant RH, Baranowski T, Rhodes T, Gutin B, Thompson WO, Carroll R, et al. Association among serum lipid and lipoprotein concentrations and physical activity, physical fitness, and body composition in young children. J Pediatr. 1993;123(2):185-92.

11. Leppanen MH, Nystrom CD, Henriksson, Leppanen, M H, Nystrom, Delisle C, Pomeroy, Ruiz, J R et al: Physical activity intensity, sedentary behavior, body composition and physical fitness in 4-year-old children: results from the ministop trial. International Journal of Obesity 2016, 40(7):1126.

12. Kolpakov V, Bespalova T, Tomilova E, Larkina NY, Mamchits E, Chernogrivova M, et al. Functional reserves and adaptive capacity of subjects with different levels of habitual physical activity. Human Physiol. 2011;37(1):93-104.

13. Specker BL, Mulligan L, Ho M. Longitudinal study of calcium intake, physical activity, and bone mineral content in infants 6-18 months of age. J Bone Miner Res. 1999;14(4):569-76.

14. Scheffler C, Ketelhut K, Mohasseb I. Does physical education modify the body composition? Results of a longitudinal study of pre-school children. Anthropologischer Anzeiger. 2007:193-201.

15. Wilson DK, Klesges LM, Klesges RC, Eck LH, Hackett-Renner CA, Alpert BS, et al. A prospective study of familial aggregation of blood pressure in young children. J Clin Epidemiol. 1992;45(9):959-69.

16. Jiménez-Pavón D, Konstabel K, Bergman P, Ahrens W, Pohlabeln H, Hadjigeorgiou C, et al. Physical activity and clustered cardiovascular disease risk factors in young children: A cross-sectional study (the IDEFICS study). BMC Med. 2013;11:172.

17. Klesges RC, Haddock CK, Eck LH. A multimethod approach to the measurement of childhood physical activity and its relationship to blood pressure and body weight. J Pediatr. 1990;116(6):888-93.

18. Sääkslahti A, Numminen P, Niinikoski H, Rask-Nissilä L, Viikari J, Tuominen J, et al. Is physical activity related to body size, fundamental motor skills, and CHD risk factors in early childhood? Pediatr Exerc Sci. 1999;11(4):327-40.

19. Sääkslahti A, Numminen P, Varstala V, Helenius H, Tammi A, Viikari J, et al. Physical activity as a preventive measure for coronary heart disease risk factors in early childhood. Scand J Med Sci Sports. 2004;14(3):143-9.

20. Damashek A, Kuhn J. Toddlers' unintentional injuries: the role of maternal-reported paternal and maternal supervision. J Pediatr Psychol. 2012:jss113.

21. Yilmaz G, Demirli Caylan N, Karacan CD. An intervention to preschool children for reducing screen time: a randomized controlled trial. Child Care Health Dev. 2015;41(3):443-9.

22. Schmidt ME, Rich M, Rifas-Shiman SL, Oken E, Taveras EM. Television viewing in infancy and child cognition at 3 years of age in a US cohort. Pediatrics. 2009;123(3):e370-5.

23. Pagani LS, Fitzpatrick C, Barnett TA, Dubow E. Prospective associations between early childhood television exposure and academic, psychosocial, and physical well-being by middle childhood. Arch Pediatr Adolesc Med. 2010;164(5):425-31.

24. Pagani LS, Fitzpatrick C, Barnett TA. Early childhood television viewing and kindergarten entry readiness. PediatrRes. 2013;74(3):350-5.

25. Lin LY, Cherng RJ, Chen YJ, Yang HM. Effects of television exposure on developmental skills among young children. Infant Behav Dev. 2015;38:20-6. PM:25544743.

26. Cheng S, Maeda T, Yoichi S, Yamagata Z, Tomiwa K, Japan Children's Study Group. Early television exposure and children’s behavioral and social outcomes at age 30 months. J Epidemiol. 2010;20(Suppl.2):S482-9.

27. Mistry KB, Minkovitz CS, Strobino DM, Borzekowski DL. Children's television exposure and behavioral and social outcomes at 5.5 years: does timing of exposure matter?. Pediatrics. 2007;120(4):762-9.

28. Irwin JD, Johnson AM, Vanderloo LM, Burke SM, Tucker P. Temperament and objectively measured physical activity and sedentary time among Canadian preschoolers. Prev Med Rep. 2015;2:598-601. PM:26844125.

29. Linebarger DL. Contextualizing video game play: the moderating effects of cumulative risk and parenting styles on the relations among video game exposure and problem behaviors. Psychol Pop Media Cult. 2015;4:375-96.

30. Byeon H, Hong S. Relationship between television viewing and language delay in toddlers: evidence from a Korea national cross-sectional survey. PLoS ONE. 2015;10:e0120663. PM:25785449.

31. Rajchanovska D, Ivanovska BZ. The impact of demographic and socio-economic conditions on the prevalence of speech disorders in preschool children in Bitola. Srp Arh Celok Lek. 2015;143:169-173. PM:26012126.

32. Linebarger DL, Barr R, Lapierre MA, Piotrowski JT. Associations between parenting, media use, cumulative risk, and children’s executive functioning. J Dev Behav Pediatr. 2014;35:367-77. PM:25007059.

33. Duch H, Fisher EM, Ensari I, Font M, Harrington A, Taromino C. Association of screen time use and language development in Hispanic toddlers: a cross-sectional and longitudinal study. Clin Pediatr (Phila). 2013;52:857-65. PM:23820003.

34. Tomopoulos S, Dreyer BP, Berkule S, Fierman AH, Brockmeyer C, Mendelsohn AL. Infant media exposure and toddler development. Arch Pediatr Adolesc Med. 2010;164:1105-11. PM:21135338.

35. Foster EM, Watkins S. The value of reanalysis: TV viewing and attention problems. Child Dev. 2010;81:368-75. PM:20331673.

36. Zimmerman FJ, Gilkerson J, Richards JA, Christakis DA, Xu D, Gray S, et al. Teaching by listening: the importance of adult-child conversations to language development. Pediatrics. 2009;124:342-9. PM:19564318.

37. Ruangdaraganon N, Chuthapisith J, Mo-Suwan L, Kriweradechachai S, Udomsubpayakul U, Choprapawon C. Television viewing in Thai infants and toddlers: impacts to language development and parental perceptions. BMC Pediatr. 2009;9:34. PM:19460170.

38. Chonchaiya W, Pruksananonda C. Television viewing associates with delayed language development. Acta Paediatr. 2008;97:977-82. PM:18460044.

39. Zimmerman FJ, Christakis DA, Meltzoff AN. Associations between media viewing and language development in children under age 2 years. J Pediatr. 2007;151:364-8. PM:17889070.

40. Miller CJ, Marks DJ, Miller SR, Berwid OG, Kera EC, Santra A, et al. Brief report: television viewing and risk for attention problems in preschool children. J Pediatr Psychol. 2007;32:448-52. PM:17012738.

41. Zimmerman FJ, Christakis DA. Children’s television viewing and cognitive outcomes: a longitudinal analysis of national data. Arch Pediatr Adolesc Med. 2005;159:619-25. PM:15996993.

42. Christakis DA, Zimmerman FJ, DiGiuseppe DL, McCarty CA. Early television exposure and subsequent attentional problems in children. Pediatrics. 2004;113:708-13. PM:15060216.

43. McKean C, Mensah FK, Eadie P, Bavin EL, Bretherton L, Cini E, et al. Levers for language growth: characteristics and predictors of language trajectories between 4 and 7 years. PLoS ONE. 2015;10.

44. Blankson A. Do hours spent viewing television at ages 3 and 4 predict vocabulary and executive functioning at age 5? Merrill-Palmer Quarterly. 2015;61:264-89.

45. Nathanson AI, Fries PT. Television exposure, sleep time, and neuropsychological function among preschoolers. Media Psychology. 2014;17:237-61.

46. Ferguson CJ, Donnellan M. Is the association between children’s baby video viewing and poor language development robust? A reanalysis of Zimmerman, Christakis, and Meltzoff (2007). Dev Psychol. 2014;50:129-37.

47. Herrmann D, Buck C, Sioen I, Kouride Y, Marild S, Molnár D, Mouratidou T, Pitsiladis Y, Russo P, Veidebaum T, Ahrens W. Impact of physical activity, sedentary behaviour and muscle strength on bone stiffness in 2–10-year-old children-cross-sectional results from the IDEFICS study. International Journal of Behavioral Nutrition and Physical Activity. 2015;12(1):112.

48. Crispim PA, Peixoto MR, Jardim PC. Risk factors associated with high blood pressure in two-to-five-year-old children. Arq Bras Cardiol. 2014;102:39-46

49. Fitzpatrick C, Pagani LS, Barnett TA. Early childhood television viewing predicts explosive leg strength and waist circumference by middle childhood. International Journal of Behavioral Nutrition and Physical Activity. 2012;9(1):87.

50. Williams HG, Pfeiffer KA, O’Neill JR, Dowda M, McIver KL, Brown WH, et al. Motor skill performance and physical activity in preschool children. Obesity (Silver Spring). 2008;16:1421-6. PM:18388895..

51. Johansson E, Hagströmer M, Svensson V, Ek A, Forssén M, Nero H, Marcus C. Objectively measured physical activity in two-year-old children–levels, patterns and correlates. International Journal of Behavioral Nutrition and Physical Activity. 2015 Jan 24;12(1):3.

52. Schmidt ME, Rich M, Rifas-Shiman SL, Oken E, Taveras EM. Television viewing in infancy and child cognition at 3 years of age in a US cohort. Pediatrics. 2009;123:e370-e375. PM:19254972.

53. Hesketh KD, Crawford DA, Abbott G, Campbell KJ, Salmon J. Prevalence and stability of active play, restricted movement and television viewing in infants. Child Care Health Dev. 2015;185(6):883-94.

54. Pagani LS, Fitzpatrick C, Barnett TA. Early childhood television viewing and kindergarten entry readiness. Pediatr Res. 2013;74:350-5. PM:23788060.

55. De Kegel A, Peersman W, Onderbeke K, Baetens T, Dhooge I, Van Waelvelde H. New reference values must be established for the Alberta Infant Motor Scales for accurate identification of infants at risk for motor developmental delay in Flanders. Child Care Health Dev. 2013;39:260-7. PM:22676145.

56. Lin LY, Cherng RJ, Chen YJ, Yang HM. Effects of television exposure on developmental skills among young children. Infant Behav Dev. 2015;38:20-6. PM:25544743.

57. Poitras VJ, Gray CE, Janssen X, Aubert S, Carson V, Faulkner G, Goldfield GS, Reilly JJ, Sampson M, Tremblay MS. Systematic review of the relationships between sedentary behaviour and health indicators in the early years (0-4 years). BMC Public Health. 2017;17(5)

58. Miller AL, Seifer R, Crossin R, Lebourgeois MK. Toddler’s self-regulation strategies in a challenge context are nap-dependent. J Sleep Res. 2015;24:279-87.

59. Gribbin CE, Watamura SE, Cairns A, Harsh JR, LeBourgeois K. The cortisol awakening response (CAR) in 2-to-4-year old children: effects of acute nighttime sleep restriction, wake time, and daytime napping. Dev Psychobiol. 2012;54:412-22.

60. Giganti F, Arzilli C, Conte F, Toselli M, Viggiano MP, Ficca G. The effect of a daytime nap on priming and recognition tasks in preschool children. Sleep. 2014;37:1087-93.

61. Plancoulaine S, Lioret S, Regnault N, Heude B, Charles MA; Eden Mother-Child Cohort Study Group. Gender-specific factors associated with shorter sleep duration at age 3 years. J Sleep Res. 2015;24:610-20

62. Vijakkhana N, Wilaisakditipakorn T, Ruedeekhajorn K, Pruksananonda C, Chonchaiya W. Evening media exposure reduces night-time sleep. Acta Paediatr. 2015;104:306-12.

63. McDonald L, Wardle J, Llewellyn CH, van Jaarsveld CH, Fisher A. Predictors of shorter sleep in early childhood. Sleep Med. 2014;15:536-40.

64. Ikeda M, Kaneita Y, Kondo S, Itani O, Ohida T. Epidemiological study of sleep habits among four-and-a-half-year-old children in Japan. Sleep Med. 2012;13:787-94.

65. Magee C, Caputi P, Iverson D. Lack of sleep could increase obesity in children and too much television could be partly to blame. Acta Paediatr. 2014;103:e27-31.

66. Lampl M, Johnson ML. Infant growth in length follows prolonged sleep and increased naps. Sleep. 2011;34:641-50.

67. Tikotzky L, De Marcas G, Har-Toov J, Dollberg S, Bar-Haim Y, Sadeh A. Sleep and physical growth in infants during the first 6 months. J Sleep Res. 2010;19:103-10.

68. Agras WS, Hammer LD, McNicholas F, Kraemer HC. Risk factors for childhood overweight: a prospective study from birth to 9.5 years. J Pediatr. 2004;145:20-25.

69. Reilly JJ, Armstrong J, Dorosty AR, Emmett PM, Ness A, Rogers I, et al. Early life risk factors for obesity in childhood: cohort study. BMJ. 2005;330:1357.

70. Bonuck K, Chervin RD, Howe LD. Sleep-disordered breathing, sleep duration, and childhood overweight: a longitudinal cohort study. J Pediatr. 2015;166:632-9.

71. Touchette E, Petit D, Tremblay RE, Boivin M, Falissard B, Genolini C, Montplaisir JY. Associations between sleep duration patterns and overweight/obesity at age 6. Sleep. 2008;31:1507-14.

72. Speirs KE, Liechty JM, Wu CF, Strong Kids Research Team. Sleep, but not other daily routines, mediates the association between maternal employment and BMI for preschool children. Sleep Med. 2014;15:1590-3.

73. Diethelm K, Bolzenius K, Cheng G, Remer T, Buyken AE. Longitudinal associations between reported sleep duration in early childhood and the development of body mass index, fat mass index and fat free mass index until age 7. Int J Pediatr Obes. 2011;6:e114-23.

74. Carter PJ, Taylor BJ, Williams SM, Taylor RW. Longitudinal analysis of sleep in relation to BMI and body fat in children: the FLAME study. BMJ. 2011;342:d2712.

75. Butte NF, Puyau MR, Wilson TA, Liu YL, Wong WW, Adolph AL, et al. Role of physical activity and sleep duration in growth and body composition of preschool-aged children. Pediatr Obes. 2016;24:1328-35.

76. Sharf RJ, DeBoer MD. Sleep timing and longitudinal weight gain in 4- and 5-year-old children. Pediatr Obes. 2014;10:141-8.

77.  Hager ER, Calamaro CJ, Bentley LM, Hurley KM, Wang Y, Black MM. Nighttime sleep duration and sleep behaviors among toddlers from low-income families: associations with obesogenic behaviors and obesity and the role of parenting. Child Obes. 2016;12:392-400.

78. Dev DA, McBride BA, Fiese BH, Jones BL, Cho H. Risk factors for overweight/obesity in preschool children: an ecological approach. Child Obes. 2013;9:399-408.

79. Jones BL, Fiese BH; STRONG Kids Team. Parent routines, child routines, and family demographics associated with obesity in parents and preschool-aged children. Front Psychol. 2014;5:374.

80. Jiang F, Zhu S, Yan C, Jin X, Bandla H, Shen X. Sleep and obesity in preschool children. J Pediatr. 2009;154:814-8.

81. Sijtsma A, Koller M, Sauer PJ, Corpeleijn E. Television, sleep, outdoor play and BMI in young children: the GECKO Drenthe cohort. Eur J Pediatr. 2015;174:631-9.

82. Watanabe E, Lee JS, Kawakubo K. Associations of maternal employment and three-generation families with pre-school children’s overweight and obesity in Japan. Int J Obes (Lond). 2011;35:945-52

83. Dieu HT, Dibley MJ, Sibbritt D, Hanh TT. Prevalence of overweight and obesity in preschool children and associated socio-demographic factors in Ho Chi Minh City, Vietnam. Int J Pediatr Obes. 2007;7:40-50.

84. Hiscock H, Scalzo K, Canterford L, Wake M. Sleep duration and body mass index in 0-7-year olds. Arch Dis Child. 2011;96:735-9.

85. Klingenberg L, Christensen LB, Hjorth MF, Zangenberg S, Chaput JP, Sjodin A, et al. No relation between sleep duration and adiposity indicators in 9-36 months old children: the SKOT cohort. Pediatr Obes. 2012;8:E14-18.

86. Gibson R, Elder D, Gander P. Actigraphic sleep and development progress of one-year-old infants. Sleep Biol Rhythms. 2012;10:77-83.

87. Fisher A, McDonald L, van Jaarsveld CHM, Llewellyn C, Fildes A, Schrempft S, et al. Sleep and energy intake in early childhood. Int J Obes. 2014;38:926-9.

88. Cardon G, De Bourdeaudhuij I, Iotova V, Latomme J, Socha P, Koletzko B, et al. Health related behaviours in normal weight and overweight preschoolers of a large pan-European sample: the ToyBox-Study. PLoS One. 2016;11:e0150580.

89. Kuzik N, Carson V. The association between physical activity, sedentary behavior, sleep, and body mass index z-scores in different settings among toddlers and preschoolers. BMC Pediatr. 2016;16:100.

90. Mindell JA, Lee C. Sleep, mood and development in infants. Infant Behav Dev. 2015;41:102-7.

91. Koulouglioti C, Cole R, Kitzman H. Inadequate sleep and unintentional injuries in young children. Public Health Nurs. 2008; 25(2):106-14.

92. Boto LR, Crispim JN, de Melo IS, Juvandes C, Rodrigues T, Azeredo P, et al. Sleep deprivation and accidental fall risk in children. Sleep Med. 2012;13:88-95.

93. Owens JA, Fernando S, Mc Guinn M. Sleep disturbance and injury risk in young children. Behav Sleep Med. 2005;3:18-31.Hager et al., 2016;

94. Hinkley T, Salmon J, Okely AD, Hesketh K, Crawford D. Correlates of preschool children’s physical activity. Am J Prev Med. 2012;42:159-67.

95. Wang H, Sekine M, Chen X, Yamagami T, Kagamimori S. Lifestyle at 3 years of age and quality of life (QOL) in first-year junior high school students in Japan: results of the Toyama Birth Cohort Study. Qual Life Res. 2008;17:257-65.

96. Chaput J-P, Gray CE, Poitras VJ, Carson V, Gruber R, Birken CS, et al. Systematic review of the relationships between sleep duration and health indicators in the early years (0-4 years). BMC Public Health 2017. 2017;17(5)

97. Reilly JJ, Kelly L, Montgomery C, Williamson A, Fisher A, McColl JH, Lo Conte R, Paton JY, Grant S: Physical activity to prevent obesity in young children: cluster randomised controlled trial. BMJ (Online) 2006, 333(7577):1041.

98. Goldfield GS, Harvey AL, Grattan KP, Temple, Goldfield, Gary S, Harvey, Alysha LJ, Kimberly P, Viviene et al: Effects of Child Care Intervention on Physical Activity and Body Composition. Am J Prev Med 2016, 51(2):225.

99. Adamo KB, Wilson S, Harvey A, Grattan KP, Naylor P-J, Temple VA, Goldfield GS: Does Intervening in Childcare Settings Impact Fundamental Movement Skill Development? Medicine and science in sports and exercise 2016, 48(5):926-932.

100. Leppänen MH, Nystrom CD, Henriksson P, Pomeroy J, Ruiz JR, Ortega FB, et al. Physical activity intensity, sedentary behavior, body composition and physical fitness in 4-year-old children: results from the ministop trial. Int J Obesity (Lond). 2016;40(7):1126-33.

101. Carson V, Tremblay MS, Chastin SFM. Cross-sectional associations between sleep duration, sedentary time, physical activity and adiposity indicators among Canadian preschool-aged children using compositional analyses. BMC Public Health 2017. 2017;17(5)

102. Carson V, Lee E-Y, Hewitt L, Jennings C, Hunter S, Kuzik N, et al. Systematic review of the relationships between physical activity and health indicators in the early years (0-4 years). BMC Public Health 2017. 2017;17(5)

103. Tremblay MS, LeBlanc AG, Carson V, Choquette L, Connor GS, Dillman C, et al. Canadian Sedentary Behaviour Guidelines for the Early Years (aged 0-4 years). Appl Physiol Nutr Metab. 2012;37:370-91.

104. Hirshkowitz M, Whiton K, Albert SM, Alessi C, Bruni O, DonCarlos L, et al. National Sleep Foundation’s updated sleep duration recommendations: final report. Sleep Health. 2015;1:233-43.

105. Paruthi S, Brooks LJ, D’Ambrosio C, Hall WA, Kotagal S, Lloyd RM, et al. Recommended amount of sleep for pediatric populations: a consensus statement of the American Academy of Sleep Medicine. J Clin Sleep Med. 2016;12:785-6.
